# Mucosal Genomics Implicate Lymphocyte Activation and Lipid Metabolism in Refractory Environmental Enteric Dysfunction

**DOI:** 10.1053/j.gastro.2021.01.221

**Published:** 2021-05

**Authors:** Yael Haberman, Najeeha T. Iqbal, Sudhir Ghandikota, Indika Mallawaarachchi, Tzipi Braun, Phillip J. Dexheimer, Najeeb Rahman, Rotem Hadar, Kamran Sadiq, Zubair Ahmad, Romana Idress, Junaid Iqbal, Sheraz Ahmed, Aneeta Hotwani, Fayyaz Umrani, Lubaina Ehsan, Greg Medlock, Sana Syed, Chris Moskaluk, Jennie Z. Ma, Anil G. Jegga, Sean R. Moore, Syed Asad Ali, Lee A. Denson

**Affiliations:** 1 Department of Pediatrics, Cincinnati Children’s Hospital Medical Center and the University of Cincinnati College of Medicine, Cincinnati, Ohio; 2 Department of Pediatrics, Sheba Medical Center, Tel-HaShomer, affiliated with the Tel-Aviv University, Israel; 3 Department of Pediatrics and Child Health, Aga Khan University, Karachi, Pakistan; 4 Department of Biological and Biomedical Sciences, Aga Khan University, Karachi, Pakistan; 5 Division of Biomedical Informatics, Cincinnati Children's Hospital Medical Center Cincinnati, Department of Computer Science, University of Cincinnati College of Engineering, Cincinnati, Ohio; 6 Department of Public Health Sciences, University of Virginia, Charlottesville, Virginia; 7 Department of Pathology and Laboratory Medicine, Aga Khan University, Karachi, Pakistan; 8 Department of Pediatrics, University of Virginia, Charlottesville, Virginia

**Keywords:** Anthropometrics, DNA Methylation, RNA Sequencing, Intestine, AKU, Aga Khan University, CRFs, conditional random forests, Ctl, control, DMRs, differentially methylated regions, DNAm, DNA methylation, EED, environmental enteric dysfunction, FDR, false discovery rate, HAZ, length/height-for-age *z* score, IFNG, interferon gamma, IGF, insulin-like growth factor, mRNAseq, messenger RNA, rDMR, regulatory DMR, SEEM, Study of Environmental Enteropathy and Malnutrition, WAZ, weight-for-age *z* score, WHZ, weight-for- length/height *z* score, WGCNA, weighted gene coexpression network analysis

## Abstract

**Background & Aims:**

Environmental enteric dysfunction (EED) limits the Sustainable Development Goals of improved childhood growth and survival. We applied mucosal genomics to advance our understanding of EED.

**Methods:**

The Study of Environmental Enteropathy and Malnutrition (SEEM) followed 416 children from birth to 24 months in a rural district in Pakistan. Biomarkers were measured at 9 months and tested for association with growth at 24 months. The duodenal methylome and transcriptome were determined in 52 undernourished SEEM participants and 42 North American controls and patients with celiac disease.

**Results:**

After accounting for growth at study entry, circulating insulin-like growth factor-1 (IGF-1) and ferritin predicted linear growth, whereas leptin correlated with future weight gain. The EED transcriptome exhibited suppression of antioxidant, detoxification, and lipid metabolism genes, and induction of anti-microbial response, interferon, and lymphocyte activation genes. Relative to celiac disease, suppression of antioxidant and detoxification genes and induction of antimicrobial response genes were EED-specific. At the epigenetic level, EED showed hyper-methylation of epithelial metabolism and barrier function genes, and hypo-methylation of immune response and cell proliferation genes. Duodenal coexpression modules showed association between lymphocyte proliferation and epithelial metabolic genes and histologic severity, fecal energy loss, and wasting (weight-for-length/height Z < -2.0). Leptin was associated with expression of epithelial carbohydrate metabolism and stem cell renewal genes. Immune response genes were attenuated by giardia colonization.

**Conclusions:**

Children with reduced circulating IGF-1 are more likely to experience stunting. Leptin and a gene signature for lymphocyte activation and dysregulated lipid metabolism are implicated in wasting, suggesting new approaches for EED refractory to nutritional intervention.

ClinicalTrials.gov, Number: NCT03588013. (https://clinicaltrials.gov/ct2/show/NCT03588013)

## Graphical abstract

**Figure:**
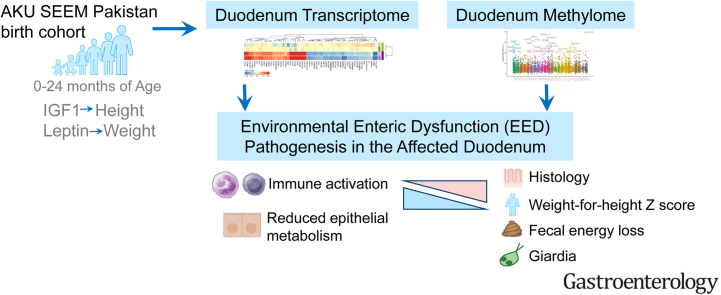

**What You Need to Know**

**Background & Context**

Environmental Enteric Dysfunction (EED) in early childhood causes significant morbidity and mortality in the developing world. Clinical and histological similarities suggest potential shared pathogenesis in the most prevalent enteropathies, celiac disease and EED.

**New Findings**

Random forest and linear regression models which accounted for initial weight and length identified associations between circulating IGF-1, ferritin, and leptin, and future growth. A core EED intestinal transcriptome was defined, emphasizing unique antimicrobial immune responses and detoxification functions relative to celiac disease. Specific immune and metabolic epigenetic and gene co-expression modules in EED were linked to histologic severity, fecal energy loss, and wasting.

**Limitations**

We were not able to include a control group of age-matched children in Pakistan with normal growth for endoscopy and mucosal genomic data, and utilized the duodenal biopsy obtained for research in the refractory EED participants to generate bulk DNA methylation and RNASeq data. Therefore, mucosal genomic results may have been limited by ethnic differences in cases and controls, and lack of single cell resolution.

**Impact**

Data suggest specific immune and metabolic pathways which may inform more effective interventions for wasting in EED.

Enteropathy in early childhood can have irreversible adverse effects on both growth and cognitive development.^1^ Environmental enteric dysfunction (EED)^2^ and celiac disease^3^ are prevalent enteropathies in this age group. Most previous studies of EED have used noninvasive stool, blood, and urine biomarkers because endoscopy is less commonly performed in the affected low-resource regions. Causative factors for EED in the diseased gut tissue have, therefore, remained incompletely defined. Previous work from Gambia using duodenal biopsy immunohistochemistry demonstrated a chronic T-cell–mediated enteropathy linked to malnutrition.^4^ In Bangladesh, recent work has defined specific EED duodenal microbiota associated with stunting, and transmission of histologic changes and weight loss to germ-free mice.^5^ Undernourished cohort from Bangladesh captured differential histone methylation in peripheral blood,^6^ and cohorts from Jamaica and Malawi characterized differential DNA methylation.^7^ However, epigenetic and transcriptional drivers of EED pathogenesis in the affected small intestine and longitudinal biomarkers to predict growth have not yet been defined.

To better characterize the longitudinal development of EED and growth failure during early childhood and to define key gut factors in children with severe EED refractory to nutritional intervention, we established the SEEM Pakistan cohort (Study of Environmental Enteropathy and Malnutrition).^8^ SEEM is an inception cohort following 416 at-risk children from birth up to 24 months of age. SEEM aimed to define longitudinal growth trajectories during the first 2 years of life and identify severe cases unresponsive to nutritional intervention that require endoscopic evaluation, define EED pathogenesis using histology, transcriptome, and epigenome (methylome) of duodenal biopsy specimens, and use data including noninvasive biomarkers collected up to 9 months of age to predict growth at 24 months.

## Methods

### Study Design

SEEM is an Aga Khan University (AKU) prospective cohort study that enrolled children at birth in Matiari, Pakistan between 2016 and 2019 undergoing evaluation for EED and growth up to 24 months of age.^8^ The SEEM cohort consisted of 416 children (365 malnourished cases and 51 well-nourished healthy controls) with a median enrollment time of 5 days after birth. Anthropometry data were collected monthly, and participants received nutritional education.^8^ Child length was measured from birth to 24 months, and we refer to length/height throughout. Blood, urine, and fecal samples were collected at 9 months of age. Nutritional intervention according to Pakistan’s Community Management of Acute Malnutrition protocol^9^ using high-calorie AchaMum therapeutic food and close monitoring was offered to 189 cases with wasting (weight-for- length/height *z* score [WHZ] < -2) at age 9–10 months up to the age of 12 months (Supplementary Figure 1). Esophagogastroduodenoscopy was performed as part of the clinical workup for 63 children with EED who did not respond to the nutritional intervention. Histology was evaluated centrally at AKU by 2 pathologists by a consensus assessment. One research duodenal biopsy specimen^10^ for molecular profiling was obtained from each of the 57 participants, and RNA for transcriptomics was available for 52. Due to ethical considerations and lack of clinical indications to perform endoscopy on well-nourished Matiari controls, biopsies from this population were not included. A prior study from Gambia demonstrated the utility of including healthy pediatric controls from the United Kingdom in defining pathogenic mechanisms in EED.^4^ We, therefore, enrolled 25 controls and 17 celiac disease subjects at the Cincinnati Children’s Hospital Medical Center. Controls were subjects who were investigated for digestive symptoms but had normal endoscopic and histologic findings. Celiac disease diagnosis was based on previously described algorithms^11^ including tissue transglutaminase auto-antibodies and characteristic histologic features. Supplementary Figure 1 illustrates the cases and controls for the transcriptomics analysis, and for the biomarker analysis. Each site’s Institutional Review Board approved the protocol and safety monitoring plan. Informed consent/assent was obtained for each participant.

### Outcomes

SEEM was designed to understand the pathophysiology, growth predictors, and potential management strategies of EED. The primary outcome was length/height-for-age *z* score (HAZ), as a measure of stunting, at 24 months of age. The secondary outcomes were WHZ, as a measure of wasting, and weight-for-age *z* score (WAZ), as a measure of underweight at 24 months, and to define genes and pathways linked to EED pathogenesis.

### Messenger RNAseq, Methylation Array, and Bioinformatic Analyses

Detailed messenger RNA (mRNAseq), methylation array, and bioinformatics methods are provided in the Supplementary Methods. Briefly, the duodenal biopsy global pattern of gene expression was determined using TruSeq mRNAseq library preparation and the Illumina platform.^12^ Genome-wide DNAm was profiled using the Illumina Infinium MethylationEPIC BeadChip platform (Illumina, Cambridge, UK; WG-317).^13^ Signed weighted gene coexpression network analysis (WGCNA) was implemented to identify modules of coexpressed genes.^14^ For each module in WGCNA, the first principal component, referred to as the *eigengene*, summarizes and represents the expression profiles of all the genes in a module. Candidate modules were identified based on the correlations between their respective module eigengenes and the phenotypic traits.

### Biomarkers

Circulating, urine, and fecal biomarkers were measured at 9 months using commercial assays (Supplementary Methods). For AKU cases that underwent endoscopy, the presence of giardia in a duodenal aspirate (n = 50) was determined (TaqMan Assay), and stool (n = 47) was collected to calculate fecal energy loss (cal/g) using bomb calorimetry.^15^

### Statistical Analysis

SEEM is reported as per the STrengthening the Reporting of OBservational studies in Epidemiology (STROBE) statement for observational cohort studies. The SEEM birth cohort study was designed to replicate the birth cohort study of 380 children conducted at AKU from 2013 to 2015 that identified EED biomarkers including IGF-1 and ferritin associated with linear growth rate at 18 months.^16^ Based on these findings, we planned to enroll 350 malnourished cases (WHZ < -2) and 50 well-nourished controls (WHZ > 0).^6^ In SEEM, 250 children with complete biomarker data at 9 months of age and growth data at 24 months of age were included in the final predictive model development, which provided 90% power to detect a slope of 0.22 for HAZ with 5% type I error. For the gene expression analysis, we planned to enroll 30 North American well-nourished controls and 50 malnourished children with EED from the SEEM cohort to provide 90% power to detect a 6-fold difference in duodenal interferon gamma (IFNG) and APOA1 gene expression with 5% type I error.^17^^,^^18^ Data were summarized descriptively as median (25th, 75th percentile) for continuous variables and frequency and percentage for categorical variables. Differences between the groups were evaluated using Wilcoxon rank sum test for continuous variables, and with chi-square test for categorical variables. The overall cohort with complete biomarker and growth data (n = 250) was randomly divided into independent training and validation groups with a 2:1 ratio. Model building was done using the training dataset, whereas the validation dataset was used to test the model performance. Conditional random forests (CRFs) analysis was performed using the training dataset to evaluate the relative importance of risk factors and log-transformed biomarkers while accounting for their correlations with a threshold of ≥ 0.5. The top predictors from CRF were used to develop the growth prediction models using linear regression. Statistical tests were conducted with 2-sided alpha level of .05. All data analyses were performed using the statistical packages SAS 9.4 (SAS Institute; Cary, NC) and R 4.0 (www.r-project.org).

#### Data availability

Data have been deposited in Gene Expression Omnibus under accession number GSE159495 (mRNAseq) and GSE157914 (methylation array chip).

## Results

### Participants

The SEEM-AKU birth cohort included 365 malnourished cases and 51 controls with adequate growth (Table 1, Supplementary Figure 1, and Supplementary Table 1) followed up to 24 months of age in Matiari, Pakistan. Positive correlations (*r* > 0.4; *P* < .001) for biomarkers^16^ measured at 9 months of age for the overall cohort were noted between IGF-1 and leptin, C-reactive protein and alpha-1-acid glycoprotein, and tumor necrosis factor α and IFNγ (Supplementary Figure 2). SEEM-AKU controls exhibited higher levels of urine creatinine, blood prealbumin, IGF1, GLP2, and leptin, and significantly reduced levels of urine Claudin15 and blood ferritin (Supplementary Table 1), in comparison with the malnourished cases. In this study, 189/365 participants with ongoing wasting (WHZ < -2) received nutritional intervention from age 9–10 months through 12 months. This resulted in a modest improvement in WAZ (mean change of 0.263 ± standard deviation [SD] of 0.704; *P* < .0001), but the infants still exhibited severe underweight (mean WAZ < -3) and stunting (mean HAZ < -2.5). Participants with ongoing wasting (median HAZ -3.2, WAZ -2.9, and WHZ of -2.2) were offered endoscopic evaluation (n = 63) around 20 months of age, and 1 research duodenal biopsy was obtained from each of the 52 for molecular profiling. Each had characteristic EED histologic features with severity scoring completed^10^ (Supplementary Table 2). We lacked indications to perform endoscopy on adequately growing local Matiari children, and, therefore, included 25 children with gastrointestinal symptoms but normal endoscopic findings, and 17 celiac cases, from Cincinnati as healthy and disease controls (Table 1). Supplementary Figure 1 illustrates the cases and controls for the molecular duodenal biopsy analysis, and for the biomarker growth model analysis.

**Table 1:** Clinical and Demographic Characteristics

Demographics	n	AKU (N = 416)	n	AKU endoscopy (N = 52)	n	Cincinnati controls (N = 25)	n	Cincinnati celiac (N = 17)
Female sex	166	40%	16	31%	11	44%	9	53%
Ethnicity (South-Asian)	416	100%	52	100%				
Ethnicity (Caucasian)					24	96%	17	100%
Nutritional intervention	189	45.43%	52	100.00%				
Age at entry (*mo*)	416	0.16 (0.07, 0.33)	52	0.2 (0.07, 0.44)				
HAZ at entry	414	-1.61 (-2.41, -0.87)	52	-1.87 (-2.81, -1.09)				
WAZ at entry	413	-1.88 (-2.76, -1.16)	51	-2.12 (-3.15, -1.63)				
WHZ at entry	349	-1.24 (-1.99, -0.54)	42	-1.62 (-1.99, -0.96)				
Biomarkers 9 mo of age^a^								
Urine creatinine (*umol/L*)	364	126.17 (88.23, 216.47)	52	122.68 (77.82, 181.94)				
CRP (*mg/dL*)	340	0.16 (0.06, 0.41)	48	0.17 (0.08, 0.57)				
Ferritin (*ng/mL*)	340	17.75 (7.00, 37.00)	48	21.50 (9.50, 54.00)				
Hemoglobin (*g/L*)	335	10.50 (9.50, 11.4)	49	10.20 (9.00, 11.30)				
IGF1 (*ng/mL*)	340	20.25 (12.44, 32.73)	50	16.87 (6.65, 27.04)				
Prealbumin (*mg/dL*)	317	14.20 (12.20, 16.70)	30	13.65 (11.80, 16.10)				
AGP (*mg/dL*)	340	101.6 (77.0, 136.0)	48	111.0 (85.5, 139.5)				
Urine Claudin15 (*ng/mL*)	364	1.35 (0.79, 2.43)	52	1.31 (0.700, 2.40)				
GLP2 (*pg/mL*)	321	1,208.9 (815.22, 1760.5)	31	1,101.1 (754.7, 1411.6)				
Leptin (*pg/mL*)	320	181.19 (102.51, 293.79)	31	180.81 (94.06, 271.91)				
Stool myeloperoxidase (*ng/mL*)	366	3,742.8 (1531, 9850)	51	3,050 (979.5, 6475)				
TNF-α (*pg/mL*)	343	64.96 (36.81 ,115.06)	50	57.175 (35.5 ,113.03)				
IFNγ (*pg/mL*)	343	7.48 (0.78 ,26.74)	50	7.995 (0.84 ,39.72)				
At the time of endoscopy								
Age (*y*)			52	1.7 (1.4, 1.9)	25	5.4 (3.8, 6.8)	17	7.3 (5.8,10)
HAZ			52	-3.2 (-3.6, -2.3)	25	0.09 (-0.51, 0.8)	17	-0.2 (-0.61, 1.17)
WAZ			52	-2.9 (-3.5, -2.6)	25	-0.08 (-1.07, 0.8)	17	-0.04 (-0.78, 0.41)
WHZ			52	-2.2 (-2.6, -1.8)				
24 mo anthropometrics								
HAZ	343	-2.33 (-3.2, -1.51)	51	-2.82 (-3.36, -2.29)				
WAZ	344	-2.25 (-2.96, -1.51)	51	-2.89 (-3.54, -2.5)				
WHZ	344	-1.31 (-2.03, -0.62)	51	-1.91 (-2.48, -1.4)				

NOTE. Data are shown as n (%) or median (25th,75th).

AGP, Alpha-1 Acid Glycoprotein; GLP2, Glucagon Like Peptide 2; TNF, tumor necrosis factor.

a Biomarkers measured at 9 mo were measured in blood unless indicated elsewhere.

### Factors Associated With Growth at 24 Months of Age

We used available clinical data and biomarkers measured at 9 months of age to predict length/height (HAZ) and weight (WAZ and WHZ) at 24 months of age. Subjects with complete biomarker and growth data (n = 250) were randomly divided into independent training (n = 166) and validation (n = 84) groups (Supplementary Table 3). Model building was performed using the training dataset, and performance was tested on the validation subset. CRFs were used to prioritize factors to test in linear regression models, with adjusted *R*^2^ used to test for overfitting (Table 2, Supplementary Table 4). For the continuous growth measures at 24 months (i.e., HAZ, WAZ, and WHZ as the primary responses), we presented *R*^**2**^, adjusted *R*^2^, and root mean square error as the primary model fitting measure. Higher HAZ around birth, and higher IGF1 and lower ferritin at 9 months, predicted higher HAZ at 24 months (adjusted *R*^2^ of 29% in the training and 32% in the validation groups; Table 2). A scatter plot for HAZ at 24 months vs IGF at 9 months is shown in Supplementary Figure 3*A* (Spearman rho = 0.305; *P* < .001). Higher WAZ around birth and higher IGF1 and leptin at 9 months predicted higher WAZ at 24 months (adjusted *R*^2^ of 25% in the training and 28% in the validation groups). A scatter plot for WAZ at 9 months vs IGF at 9 months is shown in Supplementary Figure 3*B* (Spearman rho = 0.356; *P* < .001). This may imply that IGF-1 is to some extent a surrogate marker of nutritional status. The Conditional Random Forests prioritized WHZ around birth, leptin and urine claudin15 for WHZ at 24 months. However, these factors accounted for a small amount of the variation in WHZ at 24 months (adjusted *R*^2^ of 14% in the training and 7% in the validation groups). Scatterplots of predicted vs observed values in the validation cohort for all the models built on the basis of data from the discovery cohort are shown in Supplementary Figure 3*C*. In agreement with our prior report, these data replicated circulating IGF-1 and ferritin as biomarkers to identify infants at risk for stunting (lower HAZ). However, although circulating leptin was strongly associated with wasting (lower WHZ), the multivariable model including leptin did not explain enough of the variation in weight gain to provide clinical utility. We, therefore, next tested whether the mucosal transcriptome would reveal novel immune and metabolic functions linked to wasting (lower WHZ).

**Table 2:** Models for HAZ, WAZ, and WHZ at 24 Mo of Age

CRF prioritization using training dataset (n = 166)^a^	Linear models using top 3 CRF variables	Training data (n = 166)		Validation data (n = 84)	
24 months HAZ					

	Parameter^b^	Estimate	*P*-value	Estimate	*P*-value

	Intercept	-1.965 (-2.571, -1.359)	<.001	-2.152 (-3.18, -1.125)	<.001
	HAZ at entry	0.519 (0.372, 0.667)	<.001	0.489 (0.241, 0.736)	<.001
	ln(IGF1)	0.278 (0.134, 0.422)	<.001	0.411 (0.206, 0.615)	<.001
	ln(Ferritin)	-0.120 (-0.217, -0.024)	.015	-0.216 (-0.429, -0.003)	.047
		RMSE: 0.87, *R*^2^: 31% (19%, 42%), Adjusted *R*^2^: 29% (14%, 45%)		RMSE: 1.08, *R*^2^: 35% (23%, 46%), Adjusted *R*^2^: 32% (17%, 48%)	
	24 mo WAZ				

	Parameter	Estimate	*P*-value	Estimate	*P*-value

	Intercept	-2.701 (-3.656, -1.745)	<.001	-4.246 (-5.59, -2.901)	<.001
	WAZ at entry	0.469 (0.322, 0.615)	<.001	0.408 (0.179, 0.638)	.001
	ln(IGF1)	0.247 (0.072, 0.422)	.006	0.150 (-0.075, 0.374)	.189
	ln(Leptin)	0.134 (-0.073, 0.340)	.204	0.460 (0.168, 0.752)	.002
		RMSE: 0.92, *R*^2^: 26% (15%, 37%), Adjusted *R*^2^: 25% (10%, 40%)		RMSE: 1.03, *R*^2^: 30% (19%, 42%), Adjusted *R*^2^: 28% (13%, 43%)	
	24 mo WHZ				

	Parameter	Estimate	*P*-value	Estimate	*P*-value

	Intercept	-2.674 (-3.675, -1.674)	<.001	-2.710 (-3.974, -1.445)	<.001
	WHZ at entry	0.258 (0.114, 0.402)	.001	0.179 (-0.034, 0.392)	.098
	ln(Leptin)	0.362 (0.173, 0.551)	<.001	0.326 (0.089, 0.563)	.008
	ln(urine Claudin15)	-0.148 (-0.348, 0.053)	.148	0.088 (-0.176, 0.353)	.509
		RMSE: 0.98, *R*^2^: 15% (6%, 25%), Adjusted *R*^2^: 14% (1%, 27%)		RMSE: 0.97, *R*^2^: 11% (2%, 19%), Adjusted *R*^2^: 7% (-3%, 18%)	

NOTE. Estimates and *R*^2^ are given with 95% confidence intervals.

RMSE, root-mean-square error.

a Graphs show the variable importance plots obtained with CRF for HAZ at 24 mo, WAZ at 24 mo, and WHZ at 24 mo.

b Blood IGF1, ferritin and leptin biomarkers and urine Claudin15 were obtained at 9 mo, values were analyzed using natural log transformation (ln).

### The EED Intestinal Transcriptome and Pathways

**Figure 1:**
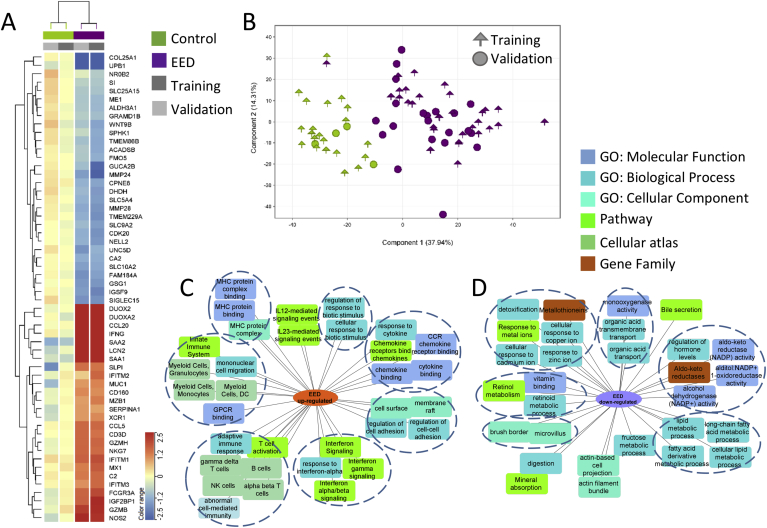
The EED intestinal transcriptome and enriched biologic pathways. The core EED transcriptome was comprised of 1,262 genes (481 down- and 781 up-regulated) differentially expressed between 31 AKU-EED malnourished cases and 21 Cincinnati well-nourished controls in the training set (FDR < 0.05 and fold change [FC] ≥1.5 using bulk RNASeq of duodenal RNA) and assessed in an independent validation set of 21 EED and 4 Ctl. Unsupervised hierarchical clustering is visualized as a heatmap in (*A*) demonstrating the averaged normalized expression in AKU-EED malnourished cases and Cincinnati well-nourished controls in the training and validation groups for the top differentially expressed genes (more detailed heatmap in Supplementary Figure 4). (*B*) Principal component analysis (PCA) using the 1,262 EED genes transcriptome (determined only using the training subset) showing separation of the AKU-EED malnourished cases and the well-nourished controls from Cincinnati in both the training and validation groups on the PC1 axis that explains 38% of the total variance in gene expression. Functional enrichment analyses of the 781 up- (*C*) and 481 down-regulated (*D*) genes between AKU-EED malnourished cases and Cincinnati well-nourished controls was performed using ToppGene/ToppCluster^34^ and was visualized using Cytoscape.^35^

We first defined the EED transcriptome in the affected duodenum. This included 1,262 genes (Figure 1, Supplementary Dataset 1) differentially expressed (FDR < 0.05 and fold change ≥ 1.5) in a training group of 31 SEEM participants with EED vs 21 healthy North American controls (Ctl; Supplementary Table 5). These differentially expressed genes were validated in an independent group of 21 EED and 4 Ctl (Figure 1, Supplementary Dataset 1). Figure 1*A* highlights the most differentially expressed genes, including up-regulation of antimicrobial *DUOX2*, *LCN2*, and *IFNG*, and down-regulation of digestion and metabolic genes *PPARGC1A*, *MMP28*, *LIPF*, and *SI*. Unsupervised hierarchal clustering using the EED transcriptome demonstrated that all Ctl and 49/52 EED from both the training and independent validation subsets clustered together (Supplementary Figure 4; chi squares on the validation set; *P* = 2.1E-5). Similarly, principal component analysis to view participants’ separation using the EED gene list showed that Ctl separated from EED in the training but also in the independent validation cohorts (Figure 1*B*). Functional enrichment analysis of the 481 down-regulated EED genes identified suppressed epithelial transporters and channels (*P* = 9.00E-10), oxidoreductases and aldo-keto reductases (*P* = 4.68E-09), lipid metabolism (*P* = 2.83E-11), genes localized to microvillus and brush border (*P* = 3.06E-07), and metallothioneins (metal-binding proteins) with antioxidant function (*P* = 5.50E-08). Up-regulated enriched EED pathways included immune activation (*P* = 7.33E-98), response to external biotic stimulus (*P* = 7.36E-76), cytokine (*P* = 7.80E-35) and interferon (*P* = 2.25E-22) signaling, alpha beta (*P* = 5.02E-77) and gamma delta (*P* = 3.09E-69) T cells, and natural killer cells (*P* = 9.23E-64) (Figure 1*C* and *D*, Supplementary Dataset 1).

### Similarities and Differences Between EED and Celiac Disease

**Figure 2:**
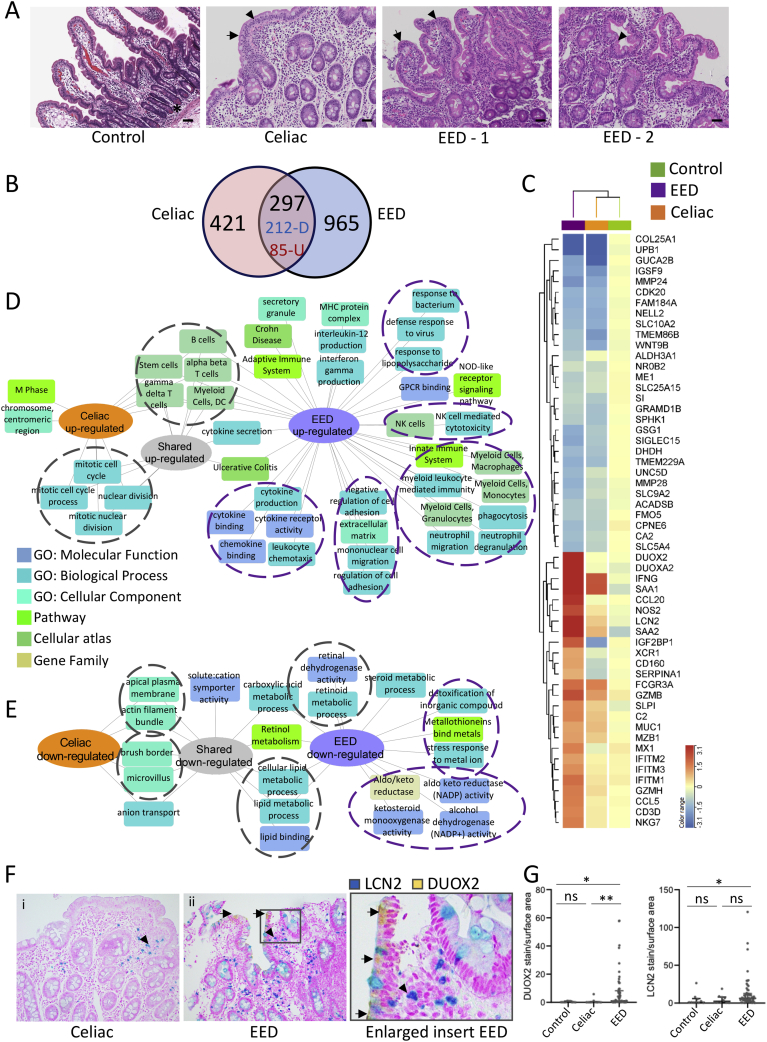
Shared and disease-specific immune and metabolic intestinal gene expression features of EED and celiac disease. (*A*) Representative hematoxylin and eosin stained duodenal biopsy specimens from a Cincinnati well-nourished control, a Cincinnati celiac disease patient (Marsh celiac disease score 3a; EED histology score of 12), a malnourished AKU-EED-1 case with EED histology score of 9, and a malnourished AKU-EED-2 case with EED histology score of 4 are shown. ∗Paneth cells in a Cincinnati well-nourished control. *Arrow* indicates villous blunting and *arrowhead* indicates intraepithelial lymphocytes in a patient from Cincinnati with celiac disease and a malnourished AKU-EED case. Bar equals 247 μm. (*B*) The Venn diagram shows the overlap between the 718 genes comprising the celiac disease transcriptome (differentially expressed genes between 17 patients from Cincinnati with celiac disease and 25 well-nourished controls from Cincinnati, FDR < 0.05 and fold change [FC] ≥ 1.5 using bulk RNASeq of duodenal RNA) and 1,262 genes comprising the EED transcriptome. This demonstrates 212 shared down- and 85 shared up-regulated genes. (*C*) Unsupervised hierarchical clustering heatmap with the top differentially expressed genes in the EED transcriptome demonstrating the averaged normalized expression across malnourished AKU-EED cases, patients from Cincinnati with celiac disease, and Cincinnati well-nourished controls. Functional enrichment analysis of the up- (*D*) and down-regulated (*E*) shared and unique genes in the EED and celiac disease transcriptomes was performed using ToppGene/ToppCluster^34^ and was visualized using Cytoscape.^35^ (*F*) Immunohistochemistry was performed using antibodies against DUOX2 (*yellow chromogen*) and LCN2 (*teal chromogen*) in a dual stain. Original magnification x200 for i & ii. (*G*) Data for the relative tissue area exhibiting staining for the analytes, normalized against the total area of tissue in each sample, are shown for controls (n = 10), celiac disease (n = 10), and EED (n = 57); Kruskal-Wallis test with Dunn multiple comparisons test; ∗∗*P* < .01; ∗*P* < .05.

Impaired growth, increased intestinal permeability, and T-cell–mediated enteropathy are shared features between celiac disease and EED,^4^ and we, therefore, included 17 celiac cases as disease controls. Representative histology for the healthy controls, celiac disease, and EED cases is shown in Figure 2*A*. Histology features used to define EED severity^10^ included villus blunting, intraepithelial lymphocytes, and Paneth cell depletion (Supplementary Table 2). The mean (SD) histology score of the EED cases was 5.7 (2.7), whereas the mean (SD) score for the celiac cases was 8.3 (4). In comparison to celiac disease, the EED cases demonstrated less pronounced intraepithelial lymphocytes and villous blunting, and more pronounced Paneth cell depletion (Supplementary Figure 5).

The celiac transcriptome included 718 genes (Figure 2, Supplementary Dataset 1) differentially expressed (FDR < 0.05 and fold change ≥ 1.5) between 17 celiac cases and 25 Ctl. A Venn diagram (Figure 2*B*, Supplementary Dataset 1) indicates the overlap between EED and celiac signatures, whereas the heat map in Figure 2*C* illustrates the expression of the core EED genes across EED, celiac, and controls. The bacterial sensor *DUOX2* and its adaptor *DUOXA2*, anti-viral defense genes (*IFITM* family), lipocalin-2 (*LCN2*), and several *CCL* chemokines were more specifically up-regulated in EED, whereas *IFNG* was up-regulated in both disorders. Shared down-regulated genes included the bile-acid transporter *SLC10A2*, carbohydrate (*SI*), lipid (*APOA1*), and retinol metabolic genes, whereas reduction of genes linked to detoxification (*ALDH3A1*), metal binding (metallothioneins family), and the aldo-keto reductase (*AKR1C*) family were specific to EED. Enrichment analyses are shown in Figure 2*D* and *E*, highlighting shared signals for up-regulation of alpha-beta and gamma-delta T lymphocytes, and for cell cycle and mitosis. More unique enrichments for EED included activation of innate responses to microbes, and cell adhesion. Shared down-regulated signals included genes linked with brush border functions, and lipid and retinol metabolism, whereas a more unique EED signal was linked with suppression of detoxification and aldo-keto reduced nicotinamide adenine dinucleotide phosphate (NADPH) reduction functions. Consistent with this, a greater level of LCN2 and DUOX2 protein staining (per stained surface area) was detected in duodenal biopsy specimens from EED subjects vs controls (Figure 2*F* and *G*, Supplementary Figure 6). Although there was some LCN2 stain detected also in celiac disease, DUOX2 staining was specific to the EED cases, and no LCN2 and DUOX2 was detected in controls. GZMB was observed in mononuclear inflammatory cells present in the lamina propria, and EED samples exhibited a higher number of granzyme-positive cells when compared with both celiac disease cases and controls (Supplementary Figure 6).

### Variation in DNAm Associated With EED Gene Expression

**Figure 3:**
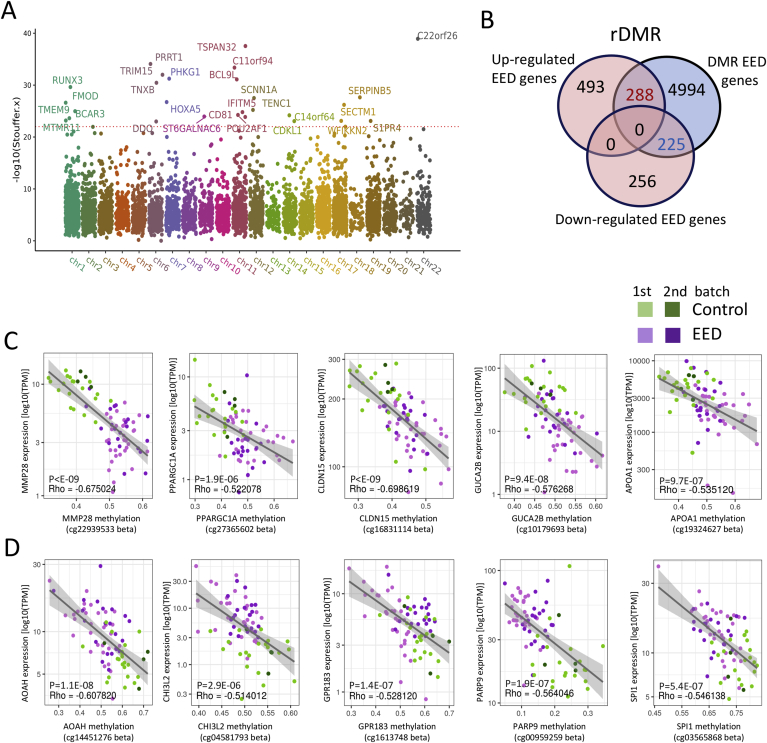
Variation in DNAm associated with expression of immune and metabolic genes in EED. Genome-wide intestinal DNAm was profiled in DNA prepared from duodenal biopsy specimens using the Illumina Infinium MethylationEPIC BeadChip platform. (*A*) A Manhattan plot is shown displaying the overlapping DMRs associated with EED in 2 methylation profile batches including 31 malnourished AKU-EED cases compared with 21 well-nourished Cincinnati controls in batch 1, and 33 malnourished AKU-EED cases compared with 9 well-nourished Cincinnati controls in batch 2, of which 12 AKU-EED cases and 5 Cincinnati controls were tested in both batches. The corrected *P* values (−log10 Stouffer) of each DMR are plotted against their respective positions on each chromosome. (*B*) The Venn diagram shows the overlap between 481 down- and 781 up-regulated genes in the EED transcriptome and DMRs highlighting 453 rDMR including genes that show evidence for both differential methylation (DM) and differential expression (DE). Beta-value methylation levels of differentially methylated points within rDMR showing a significant relationship (*P* < 1E-6) between methylation levels and expression (TPM) of specific down- (*C*) and up-regulated (*D*) genes as indicated. We highlight genes that are expressed in intestinal epithelial cells based upon a previous isolated ileal epithelial cell dataset^13^ and single-cell datasets.^22^ The *gray lines* illustrate a linear model fit, whereas rho values indicate the Spearman correlation coefficients.

Epigenetic mechanisms including DNAm mediate environmental influences on gene expression.^19^ Evidence in animal models^20^ and in humans^21^ suggests that maternal factors influence the offspring’s DNAm, and thereby traits including postnatal growth. We, therefore, analyzed genome-wide DNAm of EED and control duodenal biopsy specimens. Principal component analysis showed separation between EED and controls (Supplementary Figure 7). We identified 31,500 (between 31 EED vs 20 Ctl) and 9,102 (between 33 EED vs 9 Ctl) differentially methylated regions (DMRs) with FDR ≤ 0.01 (Supplementary Dataset 2), which when overlapped resulted in EED DMRs linked to 5,507 protein coding genes in both comparisons. A Manhattan plot (Figure 3*A*) illustrated the most significant findings linked with EED. Those included hyper-methylation in regions near genes involved in gene transcription (*HOXA/HOXB*), wound healing (*TNXB*), and epithelial adhesion (*SERPINB5*). Hypo-methylated DMR included *TSPAN32*, located in the Beckwith Wiedemann overgrowth imprinted gene domain, the transcription factor *RUNX3* involved in chromatin modifications, differentiation, and proliferation, and the anti-viral *IFITM* gene family. We then defined regulatory DMR (rDMR) that spanned genes also differentially expressed in EED (Figure 3*B*). Down-regulated genes were enriched for differential methylation (47%; 225/481) in comparison with other expressed genes (34%; 4,539/13,464; chi-square *P* < .0001). We noted a trend toward enrichment of rDMR among the up-regulated genes (37%; 288/781 vs 34%; 4,539/13,464; chi-square *P* = .07). Figure 3*C* and *D* illustrates representative differentially methylated points within rDMR, focusing on up- and down-regulated genes that were previously shown to be expressed in human ileal epithelial cells^13^ and in an epithelial single cell data set^22^ (https://singlecell.broadinstitute.org). Increased gene expression in EED and hypo-methylation was noted in *AOAH* that hydrolyzes the acyl chain to detoxify lipopolysaccharides, *CHI3L2,* and *PARP9* involved in interferon-mediated anti-viral responses. Decreased gene expression in EED coupled with hyper-methylation was noted in the mitochondria biogenesis and lipid metabolic regulator *PPARGC1A,* wound repair gene *MMP28*, and the tight junction *CLDN15* gene that was increased in the urine of EED cases (Table 1).

### Gene Expression Modules Associated With Clinical Variables

**Figure 4:**
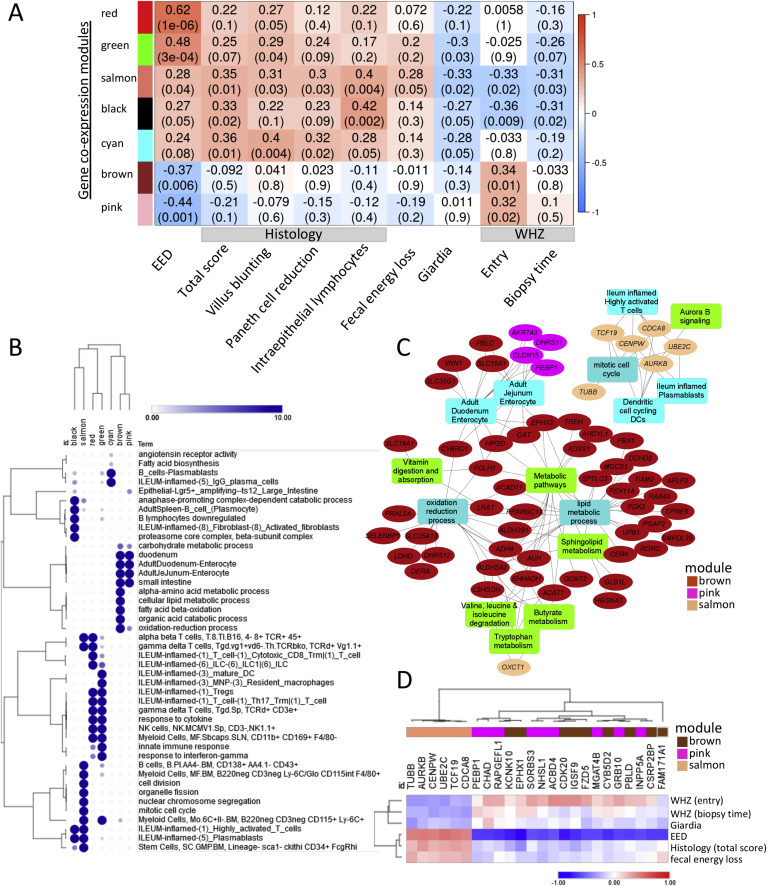
Gene coexpression modules are associated with EED diagnosis and measures of clinical and histologic severity. WGCNA was implemented to identify modules of coexpressed genes. For each module, the first principal component, referred to as the eigengene, was considered to be the module representative tested for association with phenotypic traits. (*A*) Heatmap representation of the WGCNA demonstrates gene coexpression modules (represented by module eigengenes), which were correlated with EED diagnosis (*first column*, EED), in an analysis that included 52 malnourished AKU-EED cases and 25 well-nourished Cincinnati controls and other clinical traits as shown in an analysis limited to the 52 malnourished AKU-EED cases. Seven gene coexpression modules identified based on the correlation strength with the EED diagnosis are shown, together with the results for correlations with clinical traits within the AKU-EED cases. Data are shown as the correlation coefficient and *P* value for each comparison. (*B*) A representation plot of hierarchically clustered selected top functionally enriched (FDR *P* < .05) biological processes, pathways, and cell types in each of the 7 gene coexpression modules is shown. The size of the circles and the intensity of the color is proportional to the enrichment strength. (*C*) Hub genes (*ovals*) are shown that were also differentially methylated together with functionally enriched pathways from 3 gene coexpression modules that were strongly correlated with WHZ (*salmon, pink, and brown modules*). (*D*) A heatmap of specific hub genes from (*C*) and their correlation with EED diagnosis and other clinical traits including WHZ around birth as indicated is shown.

We applied WGCNA within EED cases aiming to capture networks linked with clinical factors and biomarkers and identified 7 modules that were linked (6 with *P* < .05 and the cyan model with *P* = .08) with EED diagnosis (Figure 4*A*). The complete 13-module WGCNA heat map and gene lists including modules hub genes (top 10% with highest gene expression significance) are in Supplementary Figure 8 and Supplementary Dataset 3. The red and green modules showed the strongest positive correlation with EED diagnosis, followed by the salmon, black, and cyan modules. Those modules show enrichment for innate and adaptive immune responses, whereas the black and salmon modules were also enriched for stem cells and cell proliferation (Figure 4*B*). The presence of giardia (detected in 32/50 available duodenal aspirates) was negatively correlated with these inflammatory modules (Figure 4*A*). In contrast, the brown and pink modules showed negative associations with EED diagnosis. Those modules were linked with metabolism of amino acids and lipids, oxidation reduction, and weight (WHZ) at study entry. Modules enriched for lymphocyte and monocyte/macrophage activation and proliferation were associated with EED severity as determined using histology scoring, and more specifically with intraepithelial lymphocytes, villous blunting, and Paneth cell depletion (Figure 4*A*). Remarkably, the salmon module linked to lymphocyte and monocyte/macrophage proliferation and stem cell function was specifically correlated with fecal energy loss detected using bomb calorimetry, and WHZ (wasting) both at study entry and at the time of biopsy (Figure 4, Supplementary Figure 8). The black module also showed significant association with WHZ (wasting). Hub genes from the brown and pink modules showed significant enrichment for genes that were also differentially methylated (Supplementary Dataset 3; 47% [103/221] for brown and 66% [21/32] for pink modules vs 34%; 4,539/13,464; chi-square *P* < .03). Consistent with this, the top 15 hub genes that are also differently methylated from the pink, brown, and salmon modules and their associated pathways emphasize likely epigenetic regulation of digestive (butyrate/butanoate, tryptophan, lipid, and amino acid metabolism) and adaptive immune networks, fecal energy loss, and wasting (Figure 4*C* and *D*). Interestingly, leptin also correlated with the duodenal pink (*r* = -0.27; *P* = .05) and magenta (*r* = -0.44; *P* < .001; Supplementary Figure 8) coexpression gene modules encoding cellular metabolic functions.

## Discussion

SEEM Pakistan is a unique birth cohort that followed 416 at-risk children with varying degrees of growth impairment from birth to 24 months of age. Epigenetic and gene expression assays in the affected small intestine defined pathogenic mechanisms in children with wasting resistant to nutritional intervention. Coexpression module analysis identified associations between genes encoding lymphocyte and monocyte/macrophage proliferation and stem cell function and key histologic features including intraepithelial lymphocytes, villous blunting, and Paneth cell depletion, in conjunction with fecal energy loss and wasting. Modules enriched for immune cell proliferation and metabolic functions demonstrated a differential epigenetic signal, which correlated with wasting (WHZ) around birth and at the time of biopsy. Infants with higher circulating IGF1, and lower ferritin, at 9 months were less likely to be stunted at 24 months, providing external replication of the results from our previous EED birth cohort^16^ and another recent EED study from Bangladesh.^5^ Adjusted *R*^2^ of 32% and 28% for HAZ and WAZ, respectively, in our validation set indicated that the variations in responses can be explained fairly well, which may be useful in clinical practice and future research studies to identify children at greatest risk for stunting and associated future neurocognitive deficits. Here we further implicate leptin, previously shown to predict mortality in severely malnourished children,^19^ as a biomarker for future weight gain in a more stable population. Leptin measured at 9 months was associated with both duodenal expression of metabolic and stem cell renewal genes around 20 months, and the degree of wasting at 24 months. Collectively, these data define molecular pathways and biomarkers of EED pathogenesis, outcome, and severity.

We focused on celiac disease as a highly relevant enteropathy disease control group.^10^ Although there was substantial overlap at the molecular level between celiac and EED, we also emphasize EED-specific genes and pathways. These include up-regulation of an innate anti-microbial *DUOX2* and *LCN2* gene signature coupled with reduction of metallothioneins (*MT* family) that buffer against toxic metals, and aldo-keto NADPH–dependent reduction genes (*AKR1C* family) involved in detoxification of environmental compounds. Those features widely overlap with the intestinal Crohn’s disease transcriptome,^17^^,^^23^ suggesting similar pathogenic mechanisms involving altered gut microbiota.^5^ In fact, the antimicrobial gene signature detected in SEEM is quite consistent with the recent report of duodenal microbiota, and host defense proteins, associated with stunting in children in Bangladesh^5^ and Zambia.^24^ Similarly, genes linked with cell cycling were linked with more severe enteropathy and histologic features in the Zambia cohort as observed in our cohort.^24^ Importantly, the specificity of the antimicrobial DUOX2 staining can potentially be used to differentiate between celiac disease and EED that require different therapeutic approaches, but further studies in the undernourished areas should further confirm its use as a discriminatory biomarker between those enteropathies. Collectively these data support the potential for microbial-directed therapy to improve growth in EED. Microbiome-directed complementary feeding approaches are an active field of research.^5^^,^^25^

Gene coexpression modules regulating immune and metabolic functions in EED were linked to histologic severity, fecal energy loss, and wasting, with data supporting epigenetic regulation. Features of the EED transcriptome indicate a maladaptive gut inflammatory response, supporting results from a randomized controlled trial in Kenyan children with severe acute malnutrition in which treatment with the anti-inflammatory medication mesalazine was well-tolerated and produced modest reductions in several inflammatory markers vs placebo.^26^ We also observed suppression of metabolic pathways (part of the brown coexpression module; Figure 4*C*) with reduced butyrate, tryptophan, sphingolipid, and lipid metabolism, which were linked with wasting (WHZ). Similarly, low plasma tryptophan was recently associated with infections, chronic immune activation, and stunting.^27^ Interestingly, we observed that the presence of giardia significantly attenuated the inflammatory coexpression modules and may, therefore, play a role in the decreased response to vaccination noted in children with EED.^28^ This fascinating finding aligns with recent findings that showed reduced response to vaccination during helminth colonization in an animal model.^29^

Development, aging, diet, and gut microbes directly influence DNAm in the intestine. Promoting better nutrition and the gut microbial health through the lens of optimizing intestinal DNAm could inform therapies for EED that surmount its persistence in children and adults despite aggressive nutritional, pharmacological, and water, sanitation, and hygiene interventions and even immigration from low- to high-income countries. Our findings suggest that intestinal DNAm may provide a therapeutic target to reverse EED in children. Anthropometrics within the first month of life were strong predictors of growth at 24 months and such findings were consistent with that from the birth cohort studies in Bangladesh.^28^^,^^30^ Interestingly, wasting (WHZ) around the time of birth also showed significant association with several immune and metabolic duodenal gene coexpression modules measured around 20 months, some of which were also enriched for epigenetic DNAm modifications. Prenatal and perinatal environmental exposures^19^ that were not part of the current dataset may influence tissue DNAm^21^ and thereby traits expressed later in life including growth and inflammatory responses.^13^ Our findings linking early wasting to genes that are differentially expressed and methylated align with those previous observations and early determinates. Supplementation with folate—an essential methyl donor nutrient—is an effective adjunct therapy for persistent diarrhea in children with malnutrition.^31^ Further, intestinal stem cell–specific deletion of DNA methyltransferase 1^32^ or a diet deficient in folate and choline^33^ recapitulates several features of EED in mice. Further, the abundance of differentially methylated genes detected in our study suggests fecal intestinal epithelial cell methylation screens might be developed for EED to provide a noninvasive stool-based approached for detection and monitoring of EED, as is currently done for colorectal cancer. Additionally, healthy gut microbiota provides an endogenous source of methyl donor nutrient producers and microbiome-directed complementary feeding approaches are an active field of research.^25^ Collectively data suggest that interventions targeting epigenetic drivers of the core regulatory genes at an early time point, even prenatally, may be necessary to reverse mucosal injury and improve energy balance in EED.

Our work has several strengths because we investigated EED in a large birth cohort in Matiari, Pakistan, where children are at risk for undernutrition, and analyzed duodenal biopsy specimens from participants with wasting unresponsive to nutritional intervention defined in a prospective manner. The prospective study design afforded a unique opportunity to define the molecular basis for EED pathogenesis using state-of-the-art whole-genome methylome and transcriptome analyses of the affected gut and to characterize predictive biomarkers in independent training and validation groups. Limitations included the need to use an older group of North American healthy controls for the molecular comparisons due to lack of indications to perform endoscopy on adequately growing local Matiari controls and the use of bulk biopsies rather than single-cell separation, which would have been challenging in the setting of EED case sampling. We also lacked data for gestational age or birth weight and microbial data. Ongoing data generation and analysis, including future studies using more advanced technologies and biopsies from similar age and ethnic background may overcome some of these challenges.

## Conclusions

We defined a core EED intestinal transcriptome, emphasizing unique antimicrobial immune responses and detoxification functions relative to celiac disease. Specific gene coexpression modules regulating immune and metabolic functions in EED were linked to histologic severity, fecal energy loss, and wasting, with data supporting epigenetic regulation. Random forest and linear regression models, which accounted for initial weight and length, identified circulating IGF-1, ferritin, and leptin as informative biomarkers for future growth. Collectively, these data will inform enrollment of infants at greatest risk for future wasting and stunting into interventional trials of more targeted therapies in the future.
