# Supplementary material for: Mucosal Genomics Implicate Lymphocyte Activation and Lipid Metabolism in Refractory Environmental Enteric Dysfunction
Source: Gastroenterology. 2021 May;160(6):2055–2071.e0. doi: 10.1053/j.gastro.2021.01.221 (PMC8113748; doi:10.1053/j.gastro.2021.01.221)
Supplement: Supplementary file 1 — Supplementary Material [file mmc1.pdf]

Mucosal Genomics Implicate Lymphocyte Activation and Lipid Metabolism in Refractory Environmental Enteric Dysfunction

Short title: Environmental enteric dysfunction pathogenesis

Yael Haberman*[1,2] Najeeha T. Iqbal*[3,4], Sudhir Ghandikota[5], Indika Mallawaarachchi[6], Tzipi Braun[2], Phillip J. Dexheimer[1], Najeeb Rahman[3], Rotem Hadar[2], Kamran Sadiq[3], Zubair Ahmad[7], Romana Idress[7], Junaid Iqbal[3,4], Sheraz Ahmed[3], Aneeta Hotwani[3], Fayyaz Umrani[3], Lubaina Ehsan[8], Greg Medlock[8], Sana Syed[3,8], Chris Moskaluk[8], Jennie Z. Ma[6], Anil G. Jegga[1,5], Sean R. Moore[§8], Syed Asad Ali[§3], Lee A Denson[§1]

*Co-first authors

[§]Co-corresponding authors emails: sean.moore@virginia.edu, asad.ali@aku.edu, lee.denson@cchmc.org

[1]Department of Pediatrics, Cincinnati Children's Hospital Medical Center and the University of Cincinnati College of Medicine, Cincinnati, OH, USA.

[2]Department of Pediatrics, Sheba Medical Center, Tel-HaShomer, affiliated with the Tel-Aviv University, Israel

[3]Department of Pediatrics and Child Health, Aga Khan University, Karachi, Pakistan.

[4]Department of Biological and Biomedical Sciences, Aga Khan University, Karachi, Pakistan

[5]Department of Computer Science, Cincinnati Children's Hospital Medical Center and the University of Cincinnati College of Engineering, Cincinnati, OH, USA

[6]Department of Public Health Sciences, University of Virginia, Charlottesville, VA, USA

[7]Department of Pathology and Laboratory Medicine, Aga Khan University, Karachi, Pakistan

[8]Department of Pediatrics, University of Virginia, Charlottesville, VA, USA

Correspondence: Lee A Denson. Division of Pediatric Gastroenterology, Hepatology, & Nutrition, Cincinnati Children's Hospital Medical Center. MLC 2010, 3333 Burnet Avenue, Cincinnati, OH 45229. Tel: 513-636-7575. Fax: 513-636-558. Email: lee.denson@cchmc.org

Support for this study was provided by the Bill and Melinda Gates Foundation (OPP1144149 and OPP1138727) and NIDDK P30 DK078392 (Integrative Morphology and Gene Expression Cores)

Supplementary Material

Supplementary Methods *– page 4*

Table S1. AKU Sub-group Clinical and Demographic Characteristics *– page 17*

Table S2. EED Histology Score *– page 18*

Table S3. Characteristics of Training and Validation Groups used for the Linear Regression Models *– page 20*

Table S4. Goodness-of-fit Measures for the Linear Regression Models *– page 21*

Table S5. Characteristics of Participants used for mRNASeq and methylome Batches 1 and 2 *– page 22*

Figure S1. Study design *– page 23*

Figure S2. Correlations between Anthropometrics and Biomarkers *– page 24*

Figure S3: Scatterplots of IGF1 and anthropometrics, and of predicted vs. observed growth values in the validation cohort for all the models built on the basis of data from the discovery cohort. *– page 25*

Figure S4. Heatmap of EED Transcriptome for the AKU-EED and Cincinnati Control Training and Validation Groups. *– page 26*

Figure S5. Duodenal Histology Scores for the Cincinnati Controls and Celiac Disease Patients and the AKU-EED Participants. *– page 27*

Figure S6. Duodenal Immunohistochemistry for the Cincinnati Controls and Celiac Disease Patients and the AKU-EED Participants *– page 28*

Figure S7. PCA of the Duodenal Methylome in Batches 1 and 2 for the Cincinnati Controls and AKU-EED Participants. *– page 29*

Figure S8. Correlation between Duodenal Gene Co-expression Modules and AKU-EED Participant Characteristics. *– page 30*

Supplementary dataset 1. Differentially Expressed Genes and Functional Enrichments for the AKU EED and Cincinnati Celiac Disease Groups *(Separate excel)*

Supplementary data set 2. Differentially Methylated Regions (DMR) and Associated Genes for the AKU-EED Group. *(Separate excel)*

Supplementary data set 3. Duodenal Gene Co-expression Modules and Functional Enrichments for the AKU-EED Group. *(Separate excel)*

Supplementary Methods

Participants

SEEM Pakistan is a multi-institutional collaboration between the Aga Khan University Hospital (AKUH), Pakistan, University of Virginia (UVa), Cincinnati Children's Hospital Medical Center (CCHMC) and Washington University in St. Louis (WUSTL) in the USA, with funding by the Bill and Melinda Gates Foundation[1]. It is a prospective inception study enrolling children at birth in Matiari Pakistan between 2016 and 2019 undergoing evaluation for EED and growth measurement up to 24 months of age. To address potential sources of bias, SEEM included children around birth in a region with high prevalence of undernutrition. Study operation included establishment of a field site at Matiari, Pakistan, which is a rural district about 3-hour drive north of Karachi, Pakistan by the Department of Pediatrics and Child Health at AKUH. To over-come potential unidentified bias, clinical cohort was randomly divided to training and validation for growth model building with 2:1 ratio. This study is registered with ClinicalTrials.gov, NCT03588013.

Anthropometry data were collected monthly. Child length was measured from birth to 24 months, and we refer to length/height throughout. Blood, urine and fecal samples were collected at 9 months of age. Nutritional intervention according to Pakistan's Community Management of Acute Malnutrition protocol using high calorie AchaMun therapeutic food [520 kcal, 13g protein (10%), 29g fat (50%) and essential fatty acids (EFA)] and close monitoring was offered to 189 cases with WHZ < -2 at age 9-10 months up to the age of 12months.

The subset of malnourished children (63 children) who failed to respond to educational and nutritional interventions was evaluated by Esophagogastroduodenoscopy (EGD) at the AKU hospital to identify treatable causes of malnutrition and biopsy specimens were obtained for detailed assessment of histopathology, gene expression, and methyl-chip to better characterize the pathophysiology of EED. Histology was evaluated centrally at AKU (Table S2). Research

duodenal biopsies (second/third part) for transcriptome and methylome were taken from 57 of the 63, and RNA for transcriptomics was available for 52. Due to ethical consideration and lack of clinical indication to perform endoscopy on adequate growing local Matiari controls, we were unable to include biopsies from this population.

Cincinnati controls and celiac disease controls

We included adequate growing children from North America; 25 controls and 17 celiac subjects were recruited at Cincinnati Children's Hospital Medical Center (CCHMC). Controls were subjects who were investigated for various gastrointestinal symptoms including abdominal pain, but had normal endoscopic and histologic findings. Celiac disease diagnosis was based on previously described algorithms[2] including positive IgA autoantibodies against tissue transglutaminase (anti-TTG) and histologic features. Each site's Institutional Review Board approved the protocol and safety monitoring plan. Informed consent/assent was obtained for each participant.

EED Histology score

Histology was evaluated centrally at AKU by two AKU pathologists familiar with EED histologic features. Criteria were based on the recently published EED histology features[3] and those are detailed in Table S2. Those included Acute inflammation (score 0-3), Eosinophil infiltration (score 0-3), Chronic inflammation-lamina propria (score 0-3), intra-epithelial lymphocytes (score 0-4), Villus architecture (score 0-4), Intramucosal Brunner glands (score 0-3), Foveolar cell metaplasia (score 0-3), Goblet cell density (score 0-3), Paneth cell density (score 0-3), Enterocyte injury (score 0-3), Epithelial detachment (score 0-4).

Immunohistochemistry

Formalin fixed and paraffin embedded (FFPE) tissue samples were obtained. The AKU tissue samples were placed in tissue microarrays using a semi-automated apparatus (TMArrayer,

Pathology Devices) while whole histologic sections were used for the CCHMC samples for subsequent immunohistochemistry (IHC). IHC was performed for three analytes: Dual Oxidase 2 (DUOX2) and Lipocalin 2 (LCN2), Granzyme B (GZMB); the antibody sources, dilutions and chromogens used are listed in the Immunohistochemistry table reagents below**.** IHC was performed on robotic platforms (Ventana Discovery Ultra for DUOX2 and LCN2, Dako Autostainer for GMZB). Antigen retrieval for DUOX2 and LCN2 was performed using Cell Conditioner 1 (Ventana) for 64 minutes at 70°C, while antigen retrieval for GZMB was performed using low pH Envision Flex Target Retrieval Solution (Dako) for 20 minutes at 97 °C. The immunohistochemical stains were digitally scanned, and the area of analyte staining was quantitated by image analysis algorithms.

Immunohistochemistry table reagents

Analyte	Antibody source	Antibody catalog #	Antibody dilution	Chromogen
DUOX2	Sigma-Aldrich	MABN 787	1:200	DISCOVERY Yellow
LCN2	Sigma-Aldrich	HPA 002695	1:200	DISCOVERY Teal
GZMB	Abcam	Ab 4059	1:400	3,3'-diaminobenzidine tetrahydrochloride (DAB).

Biomarkers

Blood Cytokine/Chemokine Assays:

The MILLIPLEX MAP Human Cytokine / Chemokine panel (EMD Millipore corporation, Billerica, MA). Serum samples were diluted 1:100 in a serum matrix provided in the kit. The coating beads are pre-mixed and were used as per manufacturer instruction. Beads for nine analytes were multiplexed on magnetic beads (IFNγ, TNF-α). Beads were mixed and washed with bead diluent. Samples, and standards were incubated with capture beads at room temp for 2hrs, followed by addition of detection antibodies (steptavidin-phycoerythrin). The intensity of fluorescence is directly proportional to the amount of analyte concentration. Both Mean MFI and concentration of analytes were calculated using standard curve (10,000 -3.2 pg/ml). Plates were run on Bioplex 200 platform using exponent software.

Blood Pre-Albumin

Pre-albumin is a marker of malnutrition. A concentration less than 10 mg/dL is associated with malnutrition[4]. This test measures pre-albumin level based on immunoturbidimetric assay or nephlometry techniques. Pre-albumin is detected in blood using goat polyclonal antibody specific for human pre-albumin. Concentration of pre-albumin determined in mg/dL by measuring the turbidity of immune complex at 340nm. The distribution of intensity of the scattered light depends on the ratio of the particle size of the antigen-antibody complexes to the radiated wavelength.

Blood Alpha-1-acid Glycoprotein (AGP), Ferritin, and C-Reactive Protein (CRP)

AGP or Orosomucoid, Ferritin, and CRP were analyzed on automated biochemistry analyzer, Roche/Hitachi 902 (Basel, Switzerland). The principle of assay is the agglutination of analyte using specific antibodies present in commercial reagents. The sensitivity of AGP assay is 10mg/dL. The lower limit of detection of Ferritin is 0.1 ng/mL and for CRP is 0.001 mg/dL. All three inflammatory biomarkers were analyzed previously in Pakistani EED cohort[5].

Blood Insulin Like Growth Factor-1 (IGF-1)

IGF-I was analyzed on LIAISON Diasorin (Italy). The principle of the assay is "Chemiluminescence technology" with paramagnetic microparticle solid phase. The detection limit was 3 ng/ml[5].

Blood Glucagon Like Peptide 2 (GLP2)

GLP2 is a marker of enterocyte proliferation and regeneration. GLP2 was measured in neat serum sample using competitive inhibition ELISA (Cloud-Clone Corp. Fern Hurst Dr., Katy, TX, USA). The range of assay was 5000- 7.8 pg/ml

Blood Leptin ELISA

Leptin was measured using Human Leptin Quantikine ELISA Kit (R&D SYSTEMS Minneapolis, USA) within the range of neat to 1:5 diluted serum samples. The lower limit of detection was 7.8 pg/mL

Urine Claudin15

CLDN-15 was measured by a competitive immunoassay technique using BlueGene ELISA kit (Tianxiong Road, Pudong, Shanghai). In brief, CLDN-15 was measured in neat, 1:2, 1:5 and 1:10 diluted urine samples. The intensity of color was inversely proportional to the CLDN-15 concentration. A standard curve of CLDN-15 was plotted to quantify concentration of unknown samples.

Urine Creatinine (CR)

Urinary Creatinine was measured using competitive ELISA (BlueGene ELISA kit, Tianxiong Road, Pudong, Shanghai). Undiluted or 1:5 times diluted urine samples were used as needed. The intensity of color is inversely proportional to the Creatinine concentration. A standard curve was plotted using known concentration of creatinine standards. The concentration of unknown samples was calculated from Standard curve in µmol/L. The limit of detection of assay was 1.0 µmol/L.

Fecal Myeloperoxidase:

Fecal Myeloperoxidase is the marker of intestinal inflammation or presence of neutrophil in fecal samples, which negatively correlates with linear growth[6]. For the quantitative determination of Myeloperoxidase in feces, samples were diluted 1:100, 1:500, 1:1000, 1:2500 and 1:5000 in a PBS buffer. The clear supernatant were used in pre-coated ELISA plates (Immundiagnostik AG, Stubenwald-Allee Bensheim), plates were washed and incubated with detecting antibodies and finally developed with TMB substrate. All plates were read at dual wave length reader (Biorad, iMark), for the generation of a dose response curve with absorbance unit vs. concentration

Fecal calorimetry

In those children who underwent endoscopy at AKUH, fecal calorimetry (6200 Isoperibol Calorimeter; Parr Instrument Company, Moline, IL, USA) was performed (n=47) to obtain macronutrient specific determination of fecal energy. Caloric energy content of a fecal aliquot was calculated. All samples were run in duplicate and the final value (cal/g) was averaged. For each run, the calorimeter was calibrated using a Benzoic Acid tablet (known energy=6318

cal/g). Benzoic Acid tablet was used as a spike-in with each stool sample, and samples were run with and without spike-in and the Avg cal/g (minus Benzoic Acid) was calculated.

TAC giardia

For the subset of AKU cases that underwent endoscopic evaluation (n=50) a duodenal aspirate was obtained. Duodenal aspirates were measured for presence of giardia (TAC Assay, CT<35 was considered positive). TAC cards was developed in University of Virginia for the quantification of enteropathogen burden to understand the etiology of diarrheal disease in children living in Low-to-Middle-Income Countries (LMIC)[7]. Multicenter studies such as GEMS[8] and MAL-ED[9] leverage this platform for secondary data analyses. Our group has also published two manuscripts on how enteropathogen burden contributes in overall gut inflammation in children with EED using fecal samples collected at 6 and 9 months[10] and duodenal aspirates collected from 11 children underwent duodenal biopsy[11]. The current TAC version has 33 bacterial, 14 protozoal and 7 viral targets with additional antimicrobial resistance genes targeting gyrA, parC genes in Shigella flexneri, and mphA gene for Macrolide and, ctx-M gene for Extended Spectrum Beta-lactamase in E.coli. Duodenal aspirates were collected at the time of endoscopy. Under anesthesia before performing the biopsy procedure, an ERCP catheter was passed with one to pass 10 ml of normal saline to collect returned fluid as wash aspirate. The amount of fluid varies from child to child. The fluid sample was placed in 1.5ml of aliquot and stored at -80C degree until further workup. Briefly, total nucleic acid (TNA) was extracted from 400 μL of duodenal aspirate using an automated magnetic bead–based extractor, using Roche MagNa Pure Compact isolation kit I (Roche Life Sciences, Mannheim, Germany). All duodenal aspirates were spiked with internal controls of phocine herpes virus (PhHV; Houpt Laboratory, University of Virginia, Charlottesville, VA) and MS2 (MS2 bacteriophage; Houpt Laboratory, University of Virginia, Charlottesville, VA) as DNA and RNA targets, respectively for validation and monitor the efficiency of extraction, reverse transcription, and amplification steps. TAC protocol was performed as described previously[10].

Briefly, a total of 100 μL of TNA was eluted from the MagNa Pure analyzer, 40 μL of TNA was added with 60 μL of Agpath one-step RT PCR master mix, containing nuclease free water, 2X Agpath buffer and enzyme (Thermofisher Scientific, US), followed by loading on a TAC card. The card was sealed and ran on QuantStudio 7 Flex platform (Applied Biosystems, Thermo Fisher). A total of eight samples were run on a single card, extraction blanks (consisting of nuclease-free water, spiked with PhHV and MS2) and PCR blank (consisting of nuclease-free water only) were also ran after every 3rd card to rule out false positivity/negativity aspects. Sample was considered valid positive if 1) the sample's target Ct value was less than 35.0, 2) the reference extraction blank was negative for each target, and 3) the internal controls (PhHV, MS2) had a Ct value less than 35.0. These TAC cards were customized to detect common enteropathogens. Of the 50/52 that had TAC analyses of their duodenal aspirates; 32 (64%) were positive for giardia, 6 (12%) were positive for campylobacter, and 5 (10%) for cryptosporidium, while other pathogens showed less frequent positive results.

Transcriptome analysis

The duodenal biopsy global pattern of gene expression was determined using RNAseq on the Illumina platform as previously described[12, 13]. In short, RNA/DNA were isolated from one duodenal biopsy obtained during diagnostic esophagogastroduodenoscopy using the Qiagen AllPrep RNA/DNA Mini Kit (Qiagen, Valencia, CA). 52 of the 57 biopsies yielded sufficient RNA for the mRNAseq. mRNA-Seq utilized PolyA-RNA selection, fragmentation, cDNA synthesis, adaptor ligation, TruSeq RNA sample library preparation (Illumina, San Diego, CA), and paired-end 75bp sequencing in the CCHMC Digestive Health Center core facility. Reads were quantified by kallisto[14] using Gencode v24 as the reference genome and Transcripts per Million (TPM), including 13,464 protein-coding mRNA genes with TPM above 1 in 20% of the samples in our downstream analysis. All samples showed agreement between the gene expression (Y encoded genes and XIST) determined gender and the clinical reported gender. A total of 94 RNA-

Seq samples with median read depth of ~40M (35-48M IQR) on the Illumina platform were stratified into specific clinical sub-groups including Ctl (n=25), celiac (n=17) and EED (n=52). To over-come potential unidentified bias, transcriptomics cohort was randomly divided to 2 batches for training and validation; Ctl and EED were further stratified to training (21 Ctl and 31 EED) and independent validation groups (4 Ctl and 21 EED).

Differentially expressed genes between EED and Ctl in the training group had fold change (FC) ≥1.5 and false discovery rate (FDR) < 0.05 in both GeneSpring® software and using R package DESeq2 version 1.24.0, and importing and summarizing the kallisto output files to gene level with R package tximport version 1.12.3. ToppGene[15] and ToppCluster[16] software were used to perform Gene Set Enrichment Analyses (GSEA), and visualization of the networks was obtained using Cytoscape.v3.0.2[17]. Principal Coordinates Analysis (PCA) was performed to summarize variation in gene expression between patients, and principal components (PC) values were extracted for downstream analyses. Unsupervised hierarchical clustering using Euclidean distance metric and Ward's linkage rule was used to test for groups of rectal biopsies with similar patterns of gene expression. The RNASeq data have been deposited in GEO under accession number GSE159495.

Sample size**.** Based on previously published data for RNAseq sample size estimation, if we estimate a coefficient of variation of counts of 0.4 as was observed in 90% of the genes in a range of human studies, alpha of 0.05 and power of 0.8, a sample size of at least 20 per group is required for overall analysis of the global pattern of gene expression between groups[18]. The primary endpoint guiding our sample size estimate was anticipated based on differences in duodenal IFNγ and APOA1 gene expression between subjects with environmental enteropathy lacking infections and healthy controls. We anticipated that induction of IFNγ gene expression will be associated with a reduction in APOA1 gene expression as per our recently published paper in Crohn Disease[19] and Bragde's et al paper in celiac disease[20]. In the Crohn's study, the mean(SD) RPKM IFNγ gene expression at diagnosis was equal to 1.86(2.7) in Crohn disease and was equal to 0.33(0.38) in healthy controls. The mean(SD) RPKM APOA1 gene expression at diagnosis was equal to 927(1469) in Crohn

disease and was equal to 3012(3080) in healthy controls. We anticipated similar differences between environmental enteropathy and healthy controls in the current study. Based on these results, 30 healthy controls and 50 environmental enteropathy subjects without a specific treatable infection provided 90% power to detect such a difference with $\alpha = 0.05$.

Identifying co-expressed gene modules and intramodular hubs

Weighted gene co-expression network analysis (WGCNA) was implemented to identify modules of co-expressed genes[21]. The WGCNA framework identifies co-expressed gene clusters using pairwise correlations (eq. 1) between gene expression profiles across all the samples. We have used a signed version of the WGCNA framework to distinguish between positively and negatively correlated genes. Additionally, negatively correlated genes often belong to different biological categories and therefore should be placed in different modules. The gene co-expression similarities are converted to signed adjacency values using the power adjacency function (eq. 2). The adjacency matrix representing the connection strengths between genes is non-negative.

$$s_{ij} = \frac{1+cor(i,j)}{2} \quad (1)$$

$$a_{ij} = power(s_{ij}, \beta) = |s_{ij}|^{\beta} \quad (2)$$

where $cor(i,j)$ is the Pearson correlation coefficient between the expression profiles of a pair of genes i and j. The parameter β of the power function is usually selected based on the scale-free topology criterion. In this study, we found the optimal value of the power parameter β to be equal to 5. The adjacency matrix is then used to calculate topological overlaps (eq. 3) between each gene pair.

$$TOM_{ij}^{signed}(A) = \frac{|a_{ij} + \sum_{k\neq i,j} a_{ik} a_{kj}|}{\min(\sum_{k\neq i} a_{ik}, \sum_{k\neq j} a_{jk}) + 1 - |a_{ij}|} \quad (3)$$

The topological similarities represent the interconnectedness of two genes in a given gene co-expression network. Average linkage hierarchical clustering is implemented on TOM-based dissimilarities $(1 - TOM^{signed})$ to detect modules of strongly correlated genes across all samples.

The cluster sensitivity parameter ($deepSplit$) was set to its default value of 2 in order to identify balanced genes modules while the minimum number of genes in a module ($minClusterSize$) is set to 50 genes. For each module, the first principal component referred to as the *eigengene*, is considered to be the module representative. A module eigengene does not correspond to any particular gene but summarizes the expression levels of all the genes in a given module. Candidate modules are identified based on the strength and significance (Student's asymptotic p-value) of the respective module eigengenes with the phenotypic traits including the disease status. The gene modules could be further characterized by using functional genomics-based analyses.

We used both connectivity-based and phenotype-based significance scores to identify hub genes from within the candidate modules. The *module membership (MM)* score signifies the importance of a gene (connectivity-based) within the module and is calculated as the Pearson correlation coefficient between a gene's expression profile and the module eigengene (eq. 4)

$$MM_i^{(q)} = cor(x_i, E^{(q)}) \quad (4)$$

where x_i denotes the expression profile of a gene i and $E^{(q)}$ is the eigengene of module q. Similarly, the *gene significance (GS)* score is the correlation value between the expression vector and the clinical trait (disease diagnosis in this case) (eq. 5)

$$GS_i^T = cor(x_i, T) \quad (5)$$

where T is the clinical trait of choice. We computed $HubScore_i$ for each gene as the harmonic mean of *module membership* and *gene significance* scores (eq. 6).

$$HubScore_i = \frac{2}{\left(\frac{1}{MM_i^{(q)}} + \frac{1}{GS_i^T}\right)} \quad (6)$$

Any gene with a high hub score is not only central to a given module but also strongly correlated with disease diagnosis. Finally, we identified hub genes with strong (top 10%) *MM* and *GS* scores within each of the candidate modules. These hub genes are hypothesized to be the key driver genes associated with the disease and were used for additional downstream analyses.

Methylome analysis

RNA/DNA were isolated from duodenal biopsies obtained during diagnostic esophagogastroduodenoscopy using the Qiagen AllPrep RNA/DNA Mini Kit (Qiagen, Valencia, CA). Genome-wide duodenal-derived DNA methylation of EED and control cases was profiled using the Illumina Infinium MethylationEPIC BeadChip platform (Illumina, Cambridge, UK; WG-317) in two batches (Table S3) and each batch was analyzed separately. PCA was done as part of the quality control (QC) with no separation on PCA plot by gender after excluding sex chromosomes with separation by diagnosis observed for both batches (EED and controls, Figure S7).

Raw Illumina Infinium BeadChip data (i.e. IDAT files) were imported into R and normalized using minfi version 1.30.0[22]. Imported IDAT files were normalized using functional normalization (FunNorm)[23]. Probes located on X and Y-chromosomes and probes closer than 3 bp to a SNP were excluded. M-values (defined as the log2 ratio of 1 methylated probe intensity to the unmethylated probe intensity) were used for downstream DMPs (Differentially methylated positions) and DMRs (Differentially methylated regions) analysis in the DMRcate package, version 1.20.0[24]. DMPs were considered significant if the FDR was <=0.01. DMRs were calculated using a Gaussian kernel smoother with a bandwidth of 1000bp and a scaling factor of 2, and annotated to hg19. Identification of rDMRs: Following identification and annotation of significantly differentially methylated regions (DMRs) and differentially expressed genes or co-expression hub genes, rDMRs were defined as the overlap of both groups according to their unique gene symbol annotation.

Statistical methods

SEEM is reported as per the STROBE statement for observational cohort studies. The primary objective of this study was to identify the association of clinical risk factors and biomarkers with the HAZ at 24 months, the outcome of interest in this study. In addition, we investigated factors linked with 24 months WAZ, and WHZ at 24 months.

Sample size: we planned to enroll 350 children from 0 to 6 months with weight for length/height Z score (WHZ) <-2 at the time of enrollment and 50 children of the same age who would be growing normally, with WHZ > 0, to serve as controls. This cohort will serve as a validation set of the promising biomarkers identified in our previous Pakistani EED cohort[5], and will also test new biomarkers. The biomarkers for the proposed study were selected on the basis of their biological function and significant findings of our phase I study, where we observed association of some biomarkers with HAZ scores of children during 18 months of follow[5]. This study design allowed us to perform simple linear regression for 24 months outcome. In SEEM, 250 children with complete biomarker data were included in the final predictive model development, which ensured to detect a slope of 0.22 for HAZ with 90% power and 5% type I error.

Descriptive summary: Continuous variables were summarized using median & quartiles, and compared between cases vs controls, and biopsied cases vs non-biopsied cases using Wilcoxon rank sum test. Newborn gender was summarized using frequency and percentages and compared using Chi-square test between groups. Spearman correlation was used to describe bivariate relationships between quantitative variables.

Model development: The dataset was randomly split in to training (67% of the data, n=166) and validation (33% of the data, n=84) sets. Model building was done using the training dataset while the validation dataset was used only to test the model performance. Two sets of analyses were performed in the training set:

- Conditional random forests (CRF) model was used to rank the relative importance of biomarkers, which helps to identify the risk factors and biomarkers associated with HAZ at 24 months, WAZ at 24 months, and WHZ at 24 months.
- Linear regression model was performed with the top three variables obtained using CRF. Biomarkers were log transformed before adding in linear models. Goodness-of-fit was tested using R^2 and adjusted R^2.

Final model performance was tested by running the final model with validation dataset. All the analysis was carried out using SAS 9.4 and R 4.0 statistical software.

For the continuous growth measures at 24 months (i.e., HAZ, WAZ, WHZ as the primary responses), we presented R2, Adj R2 and RMSE (Root Mean Square Error) as the primary model fitting measures in Table 2, as sensitivity, specificity, AUC, ROC, positive/negative predictive value are not applicable here. Since the random forests (RF) analysis doesn't provide the quantitative relationship directly, we selected the most important predictors from the RF analysis and evaluated their relationship with the responses quantitatively in the linear model. Such quantitative relationship from the linear models can be used in practice to assess the value of the investigated markers such as IGF-1, ferritin, and leptin. The models were built based on training set only. The validation set was used to evaluate the consistency in model performances between training and validation sets.

Table S1. AKU Sub-group Clinical and Demographic and Characteristics

Demographics	N	AKU cases (N=365)	N	AKU controls (N=51)	P-value (Wilcoxon)	N	AKU endoscopy mRNAseq (N=52)	N	AKU without endoscopy (N=302)	P-value (Wilcoxon)
Female (n,%)	142	39%	24	47%	0.27	16	31%	124	41%	0.16
Ethnicity Sauth-Asia	365	100%	51	100%		52	100%	302	100%	
Ethnicity Cau										
Nutritional intervention (n,%)	189	51.78%		0.00%		52	100.00%	126	41.72%	
Age at entry[§] (months)	365	0.16 (0.07, 0.36)	51	0.13 (0.07, 0.33)	0.72	52	0.2 (0.07, 0.44)	302	0.16 (0.07, 0.33)	0.54
HAZ at entry	363	-1.74 (-2.52, -0.94)	51	-0.89 (-1.55, 0.14)		52	-1.87 (-2.81, -1.09)	300	-1.64 (-2.46, -0.91)	0.06
WAZ at entry	362	-2.03 (-2.83, -1.23)	51	-0.98 (-1.6, -0.31)		51	-2.12 (-3.15, -1.63)	300	-1.96 (-2.8, -1.2)	0.14
WHZ at entry	299	-1.36 (-2.05, -0.66)	50	-0.57 (-1.16, -0.16)		42	-1.62 (-1.99, -0.96)	249	-1.33 (-2.03, -0.65)	0.21
<u>*Biomarkers measures at 9 months of age</u>										
Urine Creatinine (umol/L)	317	121.59 (83.20, 220.25)	47	163.79 (105.03, 207.42)	**0.04**	52	122.68 (77.82, 181.94)	254	121.91 (83.87, 225.02)	0.47
CRP (mg/dL)	294	0.16 (0.07, 0.44)	46	0.09 (0.05, 0.23)	0.06	48	0.17 (0.08, 0.57)	236	0.15 (0.06, 0.41)	0.39
Ferritin (ng/ml)	294	18.40 (7.20, 38.20)	46	9.95 (5.50, 22.00)	**0.01**	48	21.50 (9.50, 54.00)	236	18 (7.1, 37.35)	0.20
Hemoglobin (g/L)	291	10.5 (9.40, 11.4)	44	10.70 (10.15, 11.75)	0.08	49	10.20 (9.00, 11.30)	233	10.5 (9.5, 11.4)	0.21
IGF1 (ng/ml)	296	19.4 (11.57, 31.47)	44	27.35 (19.26, 37.65)	**0.001**	50	16.87 (6.65, 27.04)	236	20.16 (12.44, 33.69)	**0.05**
pre-albumin (mg/dL)	271	14.00 (12.00, 16.6)	46	15.30 (13.70, 17.70)	**0.02**	30	13.65 (11.80, 16.10)	233	14 (12, 16.6)	0.47
Alpha-1 Acid Glycoprotein (mg/dL)	294	102.4 (77.0, 137.9)	46	94.8 (72.0, 126.0)	0.13	48	111.0 (85.5, 139.5)	236	101.8 (76.4, 136.8)	0.26
Urine Claudin15 (ng/mL)	318	1.56 (0.83, 2.58)	46	0.91 (0.73, 1.13)	**<.001**	52	1.31 (0.700, 2.40)	255	1.58 (0.83, 2.57)	0.34
Glucagon Like Peptide 2 - (pg/ml)	274	1133.6 (794.0, 1628.8)	47	1705.9 (835.37, 2740.1)	**0.001**	31	1101.1 (754.7, 1411.6)	235	1130.6 (793.62, 1607.2)	0.46
Leptin (pg/ml)	273	164.39 (95.85, 264.86)	47	279.85 (182.62, 391.54)	**<.001**	31	180.81 (94.06, 271.91)	234	163.58 (96.03, 262.33)	0.57
Stool Myeloperoxidase (ng/ml)	318	3553.5 (1463, 9750)	48	4672.3 (2079.5, 10575)	0.17	51	3050 (979.5, 6475)	256	3721 (1456, 10173)	0.58
TNF-α (pg/ml)	296	68.05 (37.06 ,115.52)	47	54.61 (36.35 ,104.88)	0.15	50	57.175 (35.5 ,113.03)	236	71.1 (37.135 ,115.39)	0.45
IFNγ (pg/ml)	296	8.7 (0.79 ,34.95)	47	1.8 (0.65 ,15.57)	0.06	50	7.995 (0.84 ,39.72)	236	8.325 (0.76 ,29.995)	0.89
<u>At the time of the biopsy</u>										
Age at biopsy (years)						52	1.7 (1.4, 1.9)			
HAZ at biopsy						52	-3.2 (-3.6, -2.3)			
WAZ at at biopsy						52	-2.9 (-3.5, -2.6)			
WHZ at at biopsy						52	-2.2 (-2.6, -1.8)			
<u>24 months endpoint anthropometrics</u>										
HAZ 24 months	295	-2.49 (-3.29, -1.71)	48	-1.49 (-2.36, -0.73)	**<0.001**	51	-2.82 (-3.36, -2.29)	233	-2.33 (-3.24, -1.59)	**0.01**
WAZ 24 months	296	-2.5 (-3.09, -1.72)	48	-0.99 (-1.72, -0.5)	**<0.001**	51	-2.89 (-3.54, -2.5)	234	-2.25 (-2.92, -1.57)	**<.001**
WHZ 24 months	296	-1.53 (-2.13, -0.81)	48	-0.32 (-0.76, 0.42)	**<0.001**	51	-1.91 (-2.48, -1.4)	234	-1.39 (-2.02, -0.68)	**<.001**

*Biomarkers measured at 9 months were measured in blood unless indicated elsewhere. [§]Entry refers to requirement around birth. AKU; Aga Khan University, HAZ; length/height-for-age Z score, WAZ; weight-for-age Z score; WHZ; weight-for-length/height Z score. Data are expressed as median (25th, 75th percentile) for all continuous variables.

Table S2. EED Histology Score

Feature	Grade	Description
acute inflammation	0	No PMNs observed, or only PMNs in lamina propria with no infiltration of epithelium by PMNs (cryptitis, villitis)
	1	1-2 foci of epithelial PMN infiltration or crypt microabscesses
	2	> 2 foci of epithelial PMN infiltration or crypt microabscesses but <50% of mucosa involved
	3	≥ 50% of mucosa involved by epithelial PMN infiltration
eosinophil infiltration	0	No increase in eosinophils (highly scattered in lamina propria, no intravillus or intercryptal space with >5 eosinophils)
	1	Increased eosinophils (intravillus or intercryptal space with >5 eosinophils) involving < 50% of mucosa, with no eosinophilic crypt microabcesses
	2	Increased eosinophils (intravillus or intercryptal space with >5 eosinophils) involving > 50% of mucosa, or up to 1 focus of eosinophilic epithelial infiltration or crypt microabcesses per mucosal fragment
	3	>2 foci of eosinophilic epithelial infiltration or crypt microabcesses in any mucosal fragment
chronic inflammation-lamina propria	0	No qualitative increase in mononuclear inflammatory cells (MIC) in lamina propria. Majority of villus bases contain ≤3 MIC across, on average.
	1	Increased MIC, based on villus base displaying 3-5 MIC across, on average.
	2	Increased MIC, based on villus base displaying 6-10 MIC across, on average.
	3	Increased MIC, based on villus base displaying >10 lymphocytes on average.
intra-epithelial lymphocytes	0	No areas observed with epithelial/lymphocyte ratio ≥20%
	1	Lymphocyte/epithelial ratio ≥20%, but <50%, in less than 50% of mucosa
	2	Lymphocyte/epithelial ratio ≥20%, but <50%, in greater than 50% of mucosa
	3	Lymphocyte/epithelial ratio ≥50% in less than 50% of mucosa
	4	Lymphocyte/epithelial ratio ≥50% in greater than 50% of mucosa
villus architecture	0	Majority of villi are >3 crypt lengths long
	1	Villi are ≤ 3 but > 2 crypt lengths long, in < 50% of mucosa.
	2	Majority of villi are ≤ 2 crypt lengths long, but > 1 crypt length long
	3	Villi absent, or ≤1 crypt length long, in < 50% of mucosa.
	4	Villi absent, or <1 crypt length long, in > 50% of mucosa.
intramucosal Brunner glands	0	None observed
	1	One or two foci, none involving more than 5 crypt bases
	2	3-5 foci, none involving more than 5 crypt bases
	3	> 5 foci, or any area of intramucosal Brunner glands involving >5 crypt bases
foveolar cell metaplasia	0	Not observed
	1	1-2 villus tips involved
	2	3-5 villus tips involved

	3	> 5 villus tips involved
goblet cell density	0	Most villi contain ≥10 goblet cells
	1	Goblet cells <10/ villus, involving < 25% of mucosa
	2	Goblet cells <10/ villus, involving 25-50% of mucosa
	3	Goblet cells <10/ villus, involving >50% of mucosa
Paneth cell density	0	≥5 Paneth cells/ crypt, on average
	1	2-4 Paneth cells/ crypt, on average
	2	<2 Paneth cell/crypt, involving <50% of crypt bases
	3	<2 Paneth cell/crypt, involving >50% of crypt bases
enterocyte injury	0	Majority of enterocytes (90%) show tall columnar morphology
	1	Enterocytes show low columnar (≤2:1 L:W ratio), cuboidal or flat morphology, in < 50% of mucosa
	2	Enterocytes show low columnar (≤2:1 L:W ratio), cuboidal or flat morphology, in > 50% of mucosa
	3	Any area of mucosal erosion/ulceration
epithelial detachment	0	Complete coverage of mucosal surface by epithelial cells
	1	Surface epithelium missing or detached from <25% of mucosa
	2	Surface epithelium missing or detached from 25-50% of mucosa
	3	Surface epithelium missing or detached from 51-75% of mucosa
	4	Surface epithelium missing or detached from >75% of mucosa

Table S3. Characteristics of Training and Validation Groups used for the Linear Regression Models.

Table S3a. Continuous Variables

	**Training cohort (n=166)**					**Validation cohort (n=84)**				
Variable	Mean	Std Dev	Q1	Median	Q3	Mean	Std Dev	Q1	Median	Q3
HAZ_at_entry[§]	-1.28	0.92	-1.89	-1.42	-0.6	-1.34	0.97	-2.04	-1.34	-0.52
WAZ_at_entry[§]	-1.60	0.97	-2.27	-1.61	-0.96	-1.73	0.99	-2.37	-1.72	-1.07
WHZ_at_entry[§]	-1.18	1.06	-2.00	-1.15	-0.46	-1.33	1.00	-1.91	-1.15	-0.64
IGF_9m	26.03	17.99	12.87	21.47	34.08	23.73	15.84	14.19	19.77	33.04
Leptin_9m	231.82	176.82	103.75	184.22	302.96	250.26	255.91	110.17	183.99	269.47
GLP_9m	1499.41	1321.88	796.95	1213.91	1785.9	1447.98	1255.18	776.55	1133.6	1702.47
Claudin15_9m	1.79	1.40	0.79	1.34	2.36	1.81	1.54	0.78	1.38	2.43
Hemoglobin_9m	10.55	1.43	9.70	10.7	11.5	10.30	1.52	9.4	10.4	11.25
MPO_9m	8812.17	16854.79	1450	3776	8550	7262.76	9934.6	1070	3200	9121.25
AGP_9m	105.58	40.18	74.57	96.77	130.37	106.97	47.24	75	99.5	130.23
Pre_Albumin_9m	14.93	3.52	12.6	14.25	16.7	14.80	3.88	12.2	14.55	17
Ferritin_9m	33.69	58.87	7.2	14.95	34.8	31.93	45.91	6.35	18.25	38.75
Creatinine_9m	173.64	138.01	91.5	120.9	204.25	200.65	156.42	101.53	155.97	231.35
CRP_9m	0.56	2.81	0.06	0.14	0.32	0.66	2.34	0.06	0.16	0.37
IFNγ _9m	146.65	413.47	0.94	9.53	38.33	110.33	418.74	0.63	2.81	15.57
TNFa_9m	196.87	349.31	37.52	67.23	115.52	163.12	343.55	31.33	63.87	100.67

Table S3b. Non-continuous Variables

	**Training cohort (n=166)**		**Validation cohort (n=84)**		P-value
Gender	Frequency	Percent	Frequency	Percent	0.243
Male	98	59.04	56	66.67	
Female	68	40.96	28	33.33	
Nutritional Intervention	Frequency	Percent	Frequency	Percent	0.211
No	89	53.61	52	61.90	
Yes	77	46.39	32	38.10	

[§]Entry refers to requirement around birth.

Table S4. Goodness-of-fit Measures for the Linear Regression Models.

**4a. HAZ model***				
Variable Added	Training data (n=166)		Validation data (n=84)	
	R^2	Adj R^2	R^2	Adj R^2
Initial HAZ	0.20	0.20	0.16	0.15
**ln(IGF1)	0.28	0.27	0.30	0.31
ln(Ferritin)	0.31	0.29	0.35	0.32

*R^2 and adjusted R^2 are shown for addition of each factor to the model. **ln refers to natural log transformation

**4b. WAZ model***				
Variable Added	Training data (n=166)		Validation data (n=84)	
	R^2	Adj R^2	R^2	Adj R^2
Initial WAZ	0.18	0.17	0.11	0.10
**ln(IGF1)	0.25	0.24	0.22	0.20
ln(Leptin)	0.26	0.25	0.30	0.28

* R^2 and adjusted R^2 are shown for addition of each factor to the model. **ln refers to natural log transformation

**4c. WHZ model***				
Variable Added	Training data (n=166)		Validation data (n=84)	
	R^2	Adj R^2	R^2	Adj R^2
Initial WHZ	0.07	0.06	0.02	0.01
**ln(Leptin)	0.14	0.13	0.10	0.08
ln(Claudin15)	0.15	0.14	0.11	0.07

*R^2 and adjusted R^2 are shown for addition of each factor to the model. **ln refers to natural log transformation

Table S5a. Characteristics of Participants used for mRNASeq Batches 1 and 2

	Batch 1		Batch 2	
	EED (n=31)	Ctl (n=21)	EED (n=21)	Ctl (n=4)
Age at biopsy (years)	1.5(1.2,1.7)	5.4(3.8, 6.8)	1.9(1.8,1.9)	5.9(4.5, 6.7)
HAZ at biopsy	-2.9(-3.5,-2)	0.1(-0.5,0.9)	-3.3(-3.7,-2.7)	0(-0.8,0.7)
WAZ at biopsy	-2.9(-3.4,-2.5)	0.1(-1.2,0.8)	-3.2(-3.6,-2.7)	-0.4(-1.1,0.5)
#WHZ at biopsy	-2.2(-2.7,-1.8)		-2.2(-2.6,-1.8)	

Data are expressed as median (25th, 75th percentile).

Table S5b. Characteristics of Participants used for Methyl-chip Batches 1 and 2

	Batch 1		Batch 2	
	EED (n=31)	*Ctl (n=20)	^EED (n=33)	**Ctl (n=9)
Age at biopsy (years)	1.5(1.2,1.7)	5.3(3.7, 6.8)	1.8(1.7,1.9)	6.7(5, 6.8)
HAZ at biopsy	-2.9(-3.5,-2)	0.1(-0.5,1.0)	-3.3(-3.7,-2.5)	0(-0.5,0.5)
WAZ at biopsy	-2.9(-3.4,-2.5)	0.1(-1.2,0.8)	-3.0(-3.6,-2.6)	-0.1(-1.1,0.4)
WHZ at biopsy	-2.2(-2.7,-1.8)		-2.1(-2.6,-1.8)	

*1 Ctl from the mRNAseq group was excluded from methyl-chip
^12 EED from batch 1 were rerun on batch 2 for methyl-chip
**5 Ctl from batch 1 were rerun on batch 2 for methyl-chip
#WHZ is calculated for children under 5 years
Data are expressed as median (25th, 75th percentile).

Supplementary Figures

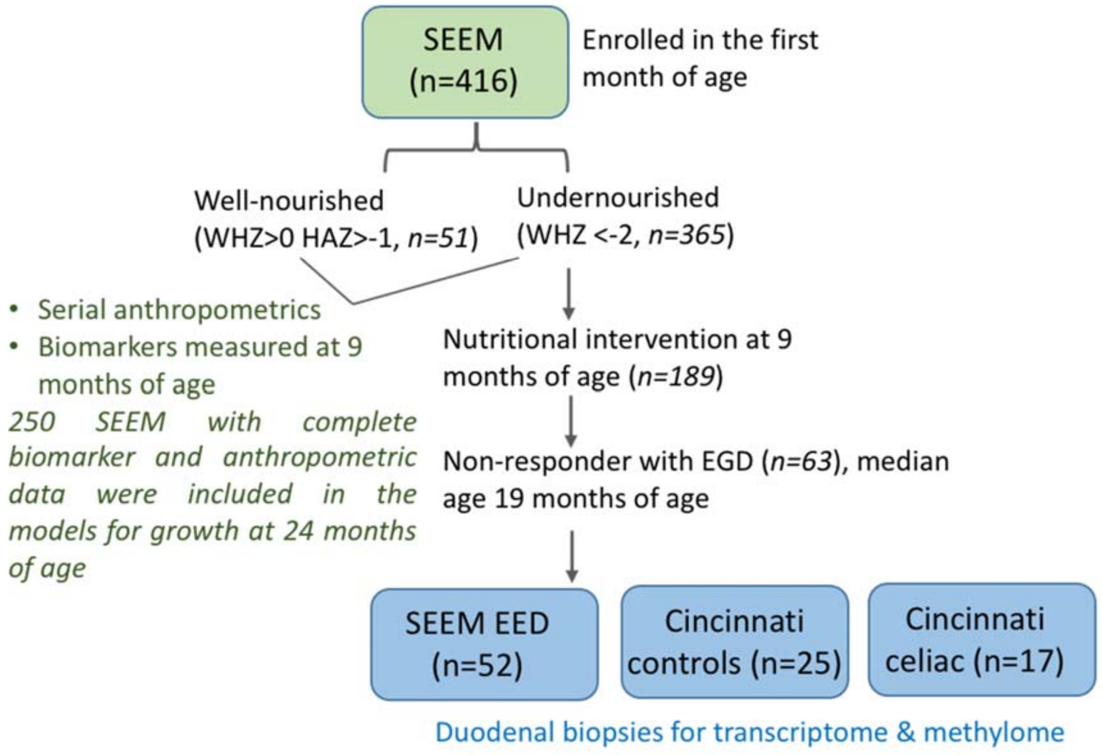

Figure S1. Cohort design. Schematic illustration of the cases and controls included in the non-invasive biomarker growth model analysis and duodenal biopsy molecular genomics analysis. EGD: Esophagogastroduodenoscopy

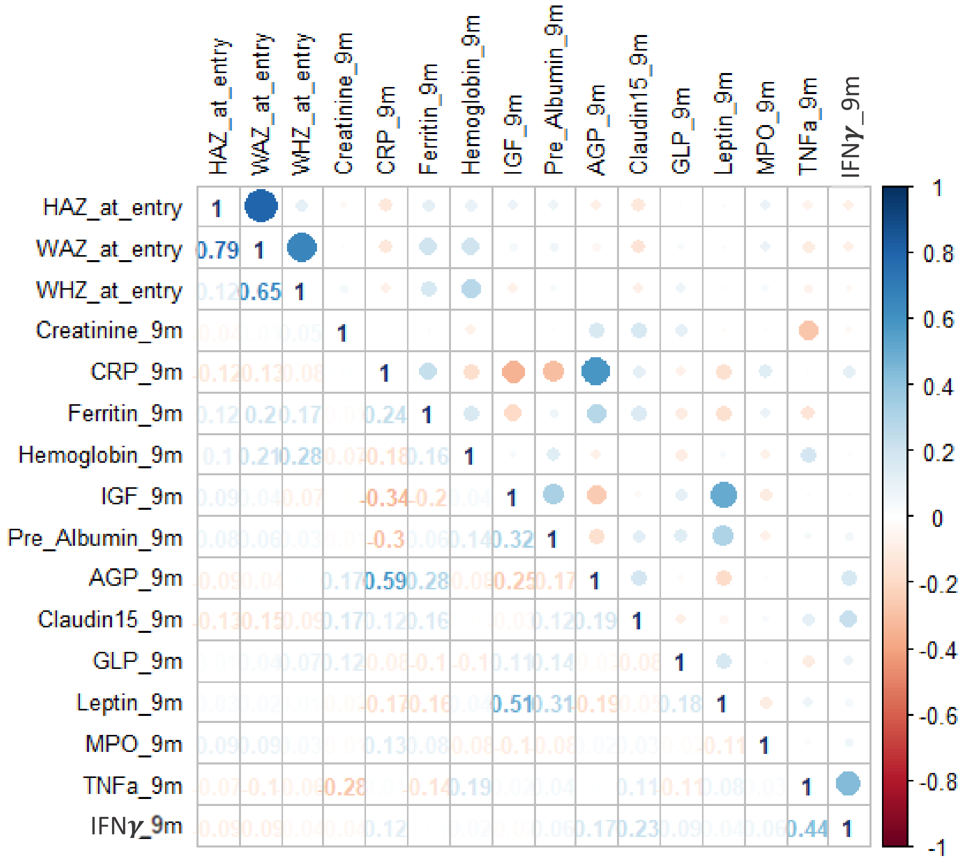

Figure S2. Correlations between Anthropometrics and Biomarkers. Correlation plot showing Spearman r values between anthropometrics at study entry and biomarkers measured at nine months of age. Blue color indicates positive correlation and red color indicates negative correlation, and the size of the circle indicates the level of correlation; the larger the circle, the more correlated the variables are. Significant Spearman correlations ($p<0.01$) include AGP and CRP ($r=0.59$), leptin and IGF ($r=0.51$), TNF-α and IFNγ ($r=0.44$), pre-albumin and IGF ($r=0.32$), pre-albumin and leptin ($r=0.31$), CRP and IGF ($r=-0.34$), CRP and pre-albumin ($r=-0.30$), and TNF-α and Creatinine ($r=-0.28$).

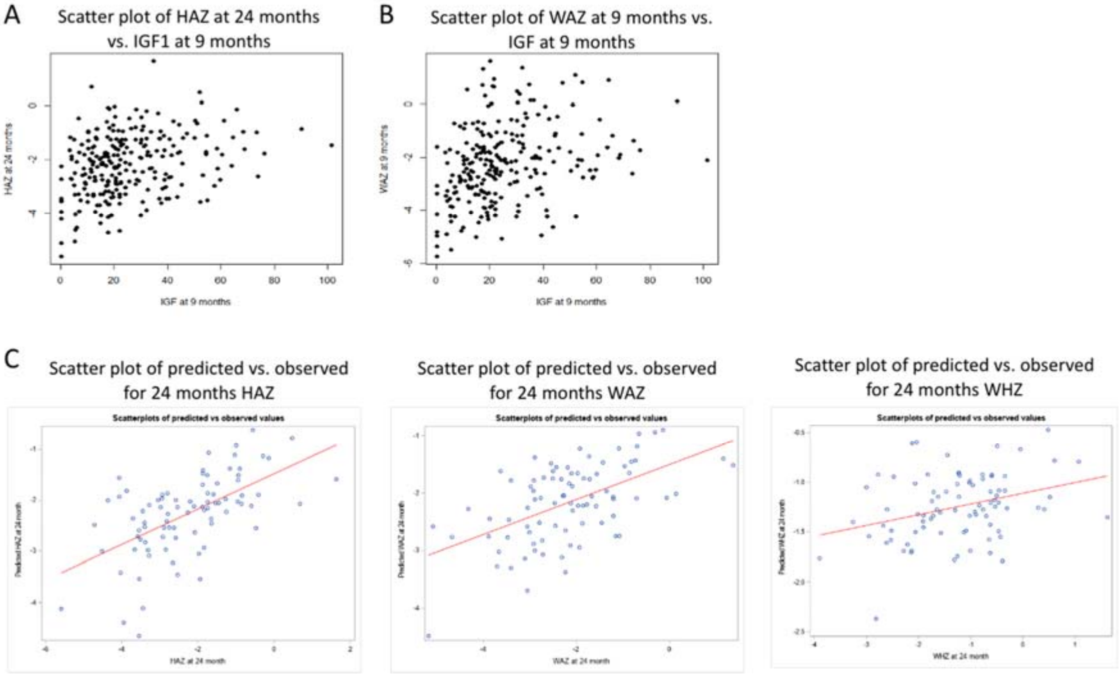

Figure S3: Scatterplots of IGF1 and anthropometrics, and of predicted vs. observed growth values in the validation cohort for all the models built on the basis of data from the discovery cohort. A. Scatterplot of HAZ at 24 months vs. IGF at 9 months with Spearman's rho = 0.305 (p-value < 0.001). **B.** Scatterplot of WAZ at 9 months vs. IGF at 9 months with Spearman's rho = 0.356 (p-value < 0.001). **C.** The validation set (n=84) is used to evaluate the consistency in model performances between training (n=166) and validation sets (n=88). Predicted vs. observed value in the validation cohort are shown for HAZ, WAZ, and WHZ at 24 months as indicated.

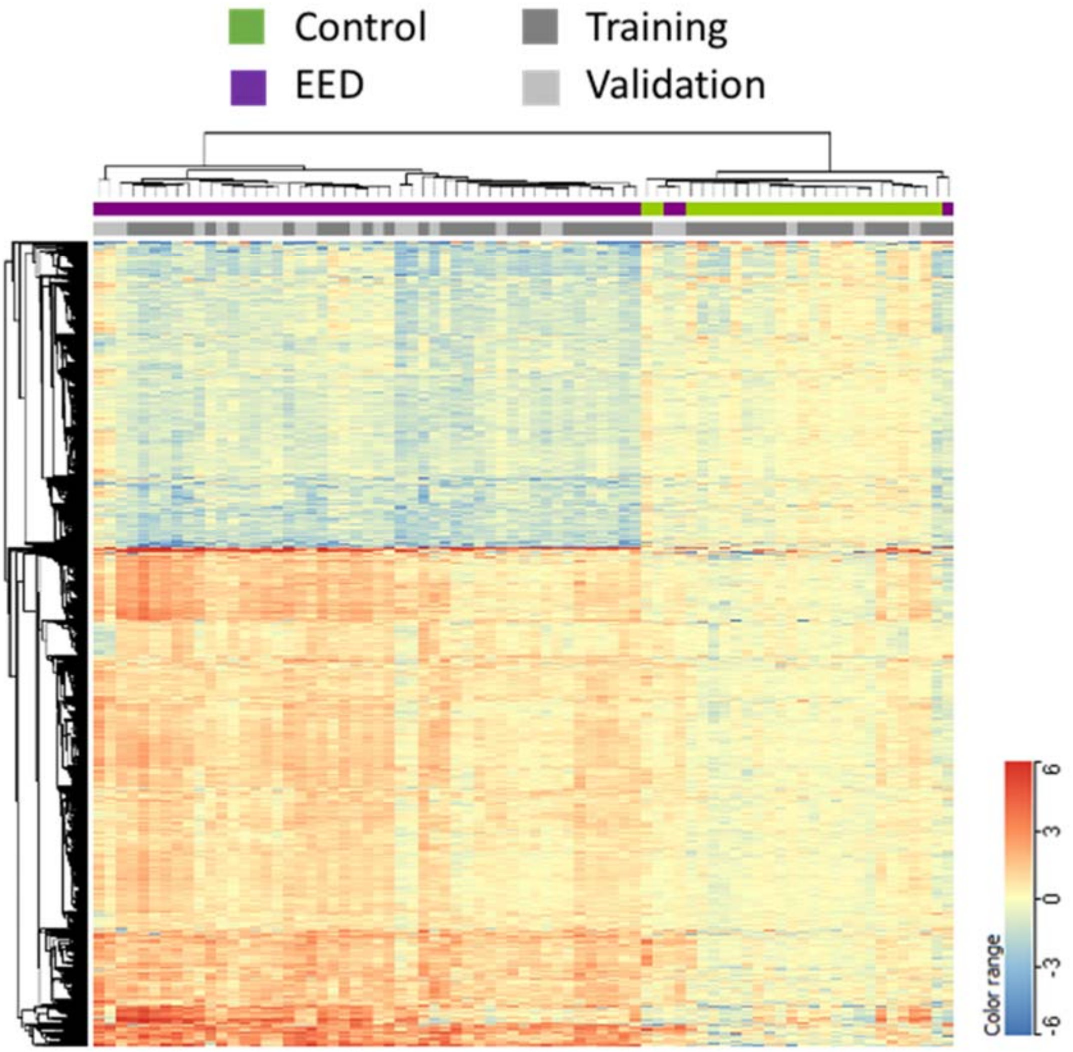

Figure S4. Heatmap of EED Transcriptome for the AKU-EED and Cincinnati Control Training and Validation Groups. The EED transcriptome is comprised of 1262 genes (481 down- and 781 up-regulated genes) differentially expressed between 31 AKU-EED and 21 Cincinnati controls (Ctl) (training set, FDR<0.05 and fold change (FC) ≥1.5) and was assessed in an independent validation subset of 21 EED and 4 Ctl. Unsupervised hierarchical clustering is visualized as a heat map in demonstrating normalized expression in EED and controls in both the training and validation groups. Above the heat map, individual Control (green) and EED (purple) samples are indicated. Training set samples are indicated by dark grey and validation set samples by light grey. Clustering defined 2 main dendrogram branches, where all training (n=21/21) and validation (n=4/4) Ctl clustered on the right and 30/31 training and 19/21 validation AKU-EED cases clustered on the left (Chi squares on the validation set, p=2.1E-5).

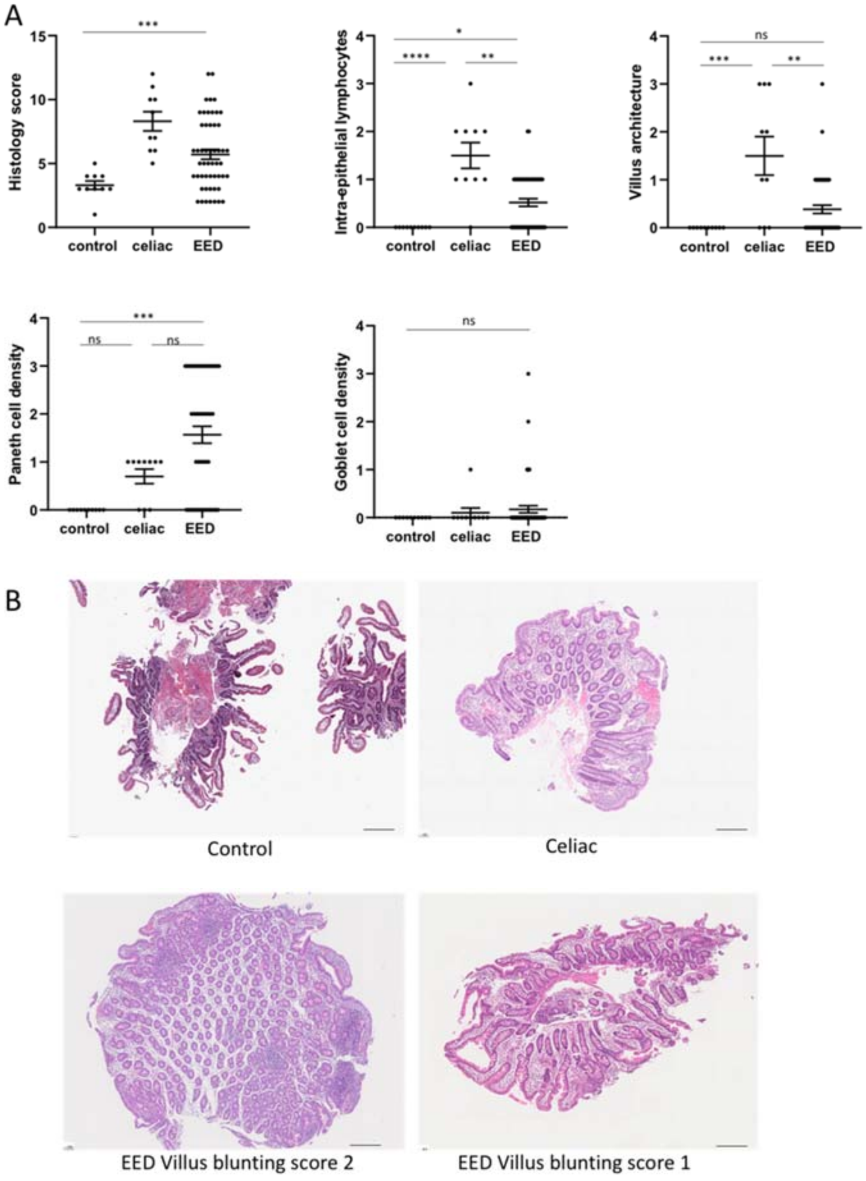

Figure S5. Duodenal Histology Scores for the Cincinnati Controls and Celiac Disease Patients and the AKU-EED Participants. A. Cincinnati control (n=10), Celiac Disease (n=10), and AKU-EED (n=52) duodenal biopsies were read centrally by two AKU pathologists using the EED histology scoring system as in Table S2. All controls were scored as zero for the four characteristic features of EED shown, with the AKU-EED duodenal biopsies exhibiting an intermediate score between celiac disease and controls for intra epithelial lymphocytes and villus architecture, and higher scores for reduced Paneth cell density. Differences between groups were tested using Kruskal-Wallis with Dunn's multiple comparisons test, *p<0.05, **p<0.01, ***p<0.001. **B.** Representative images are shown illustrating villous blunting in celiac disease and EED cases. The bar represents 1000 microns.

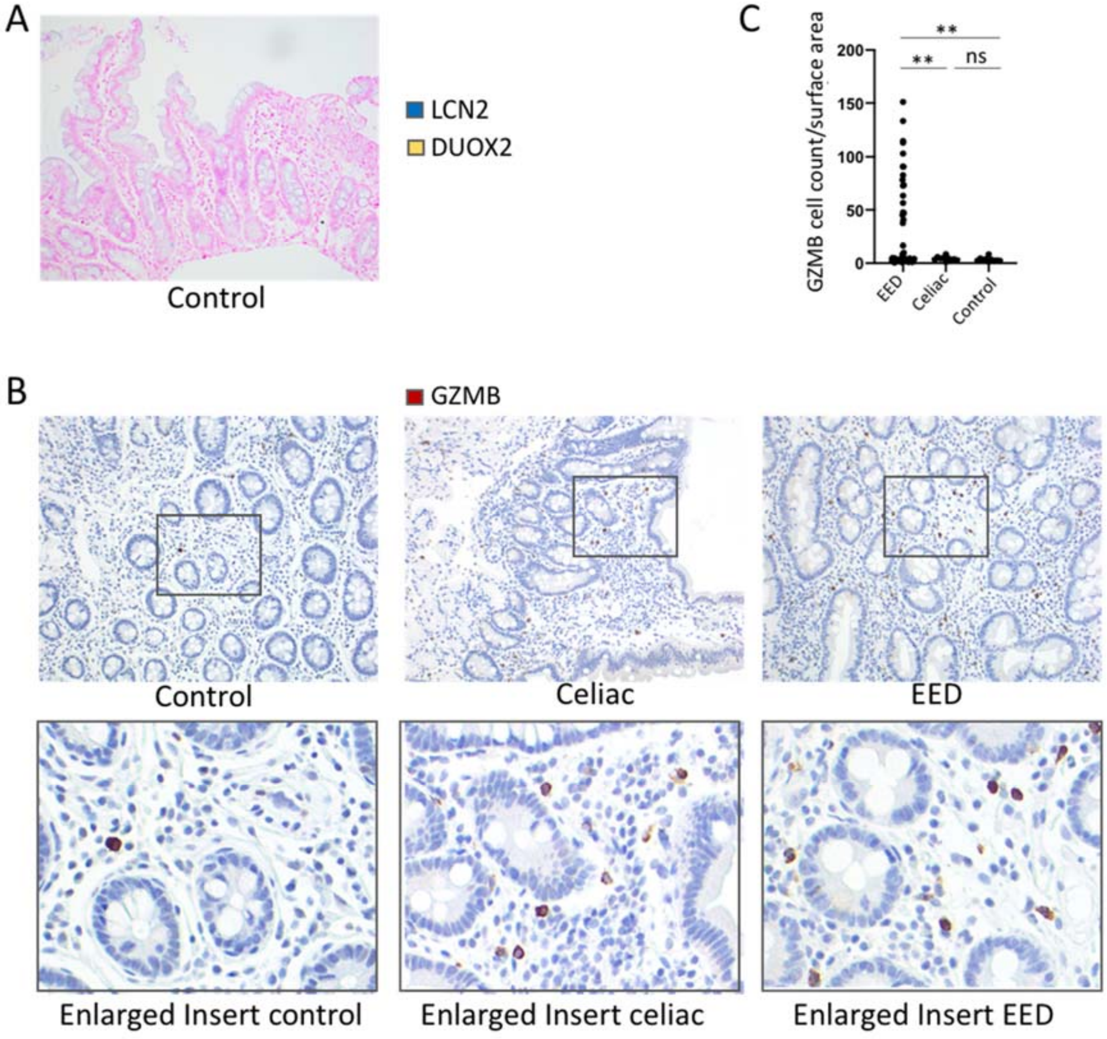

Figure S6. Duodenal Immunohistochemical analysis for the Cincinnati Controls and Celiac Disease Patients and the AKU-EED Participants. Immunohistochemistry was performed using antibodies against DUOX2 (yellow chromogen) and LCN2 (teal chromogen) in a dual stain, and against GNZB (brown chromogen) in a separate single stain. **A.** Immunohistochemical analysis for LCN2 and DUOX2 in control show no staining. Original magnification x200. **B.** Immunohistochemical analysis for GZMB in control, Celiac Disease, and EED. Original magnification x200, and the inlet area for each image is shown. **C.** Data for the GZM cell count normalized against the total area of tissue in each sample, are shown for controls (n=10), celiac disease (n=10), and EED (n=57). Poisson regression, sample type change from EE to celiac expected count decreases by 88% and from EED to control by 89%, **p<0.01.

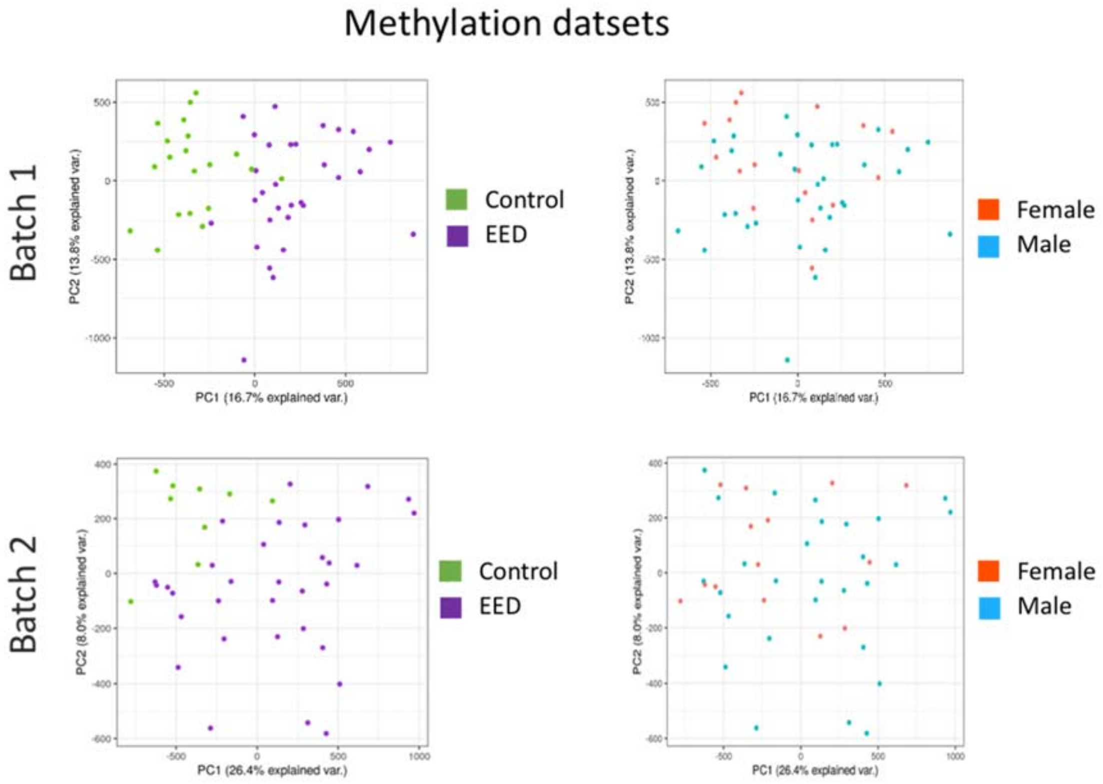

Figure S7. PCA of the Duodenal Methylome in Batches 1 and 2 for the Cincinnati Controls and AKU-EED Participants. Genome-wide duodenal-derived DNA methylation of AKU-EED and Cincinnati control cases was profiled using the Illumina Infinium MethylationEPIC BeadChip platform (Illumina, Cambridge, UK; WG-317) in two batches (Table S3) and each batch was analyzed separately. Unsupervised principal component analysis (PCA) to 2 components (PC1 and PC2) shows separation by diagnosis in both batches and no separation by gender as expected after excluding XY genes.

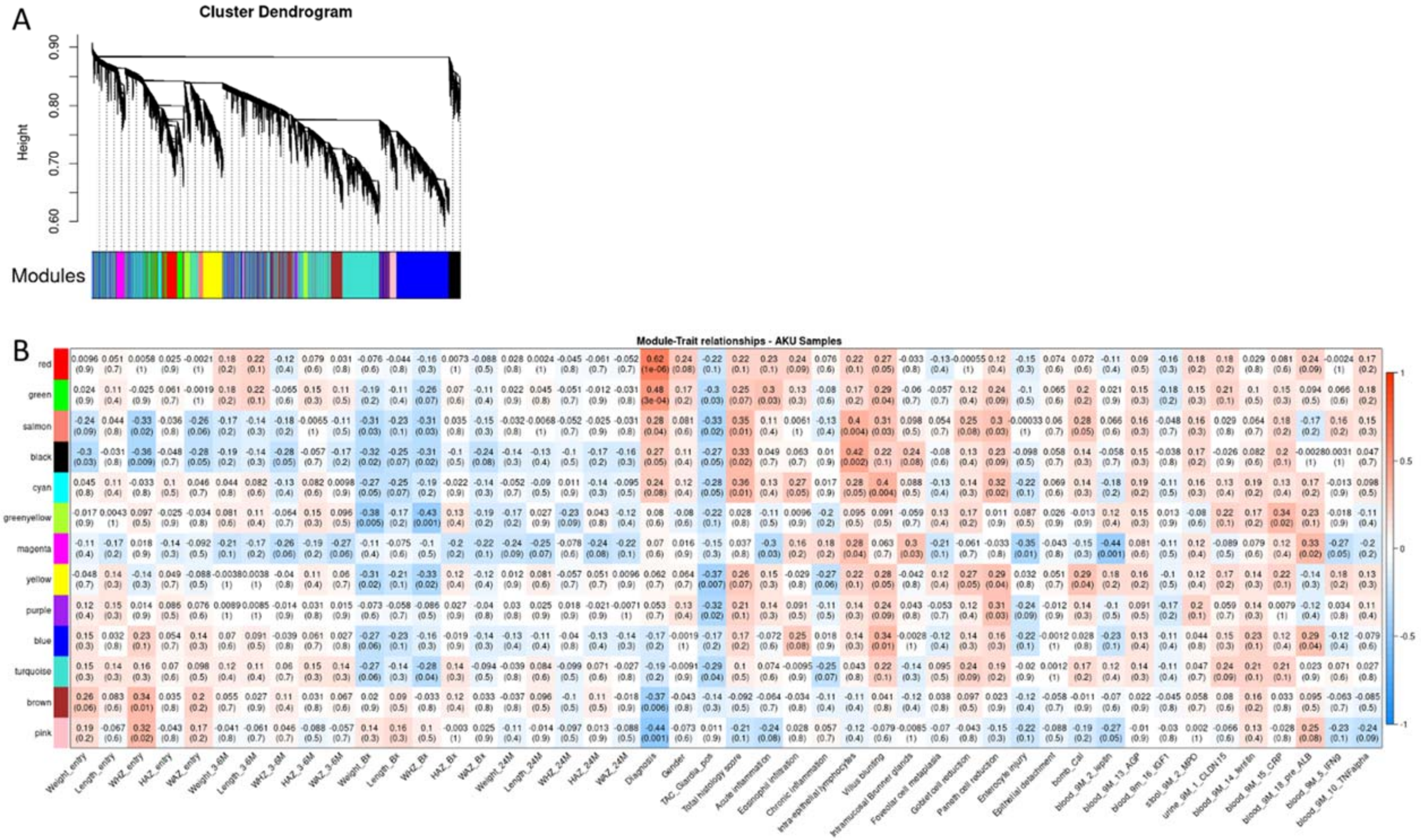

Figure S8. Correlation between Duodenal Gene Co-expression Modules and AKU-EED Participant Characteristics. A. Cluster dendrogram of the WGCNA analyses. **B**. Heat map representation of the weighted gene co-expression network analyses showing gene module eigengenes correlated with EED diagnosis and other traits, and the Pearson r and p values are indicated. M; months, Bx; time of duodenal biopsy.

References

1. Iqbal NT, Syed S, Sadiq K, et al. Study of Environmental Enteropathy and Malnutrition (SEEM) in Pakistan: protocols for biopsy based biomarker discovery and validation. BMC Pediatr 2019;19:247.
2. Scanlon SA, Murray JA. Update on celiac disease - etiology, differential diagnosis, drug targets, and management advances. Clin Exp Gastroenterol 2011;4:297-311.
3. Liu TC, VanBuskirk K, Ali SA, et al. A novel histological index for evaluation of environmental enteric dysfunction identifies geographic-specific features of enteropathy among children with suboptimal growth. PLoS Negl Trop Dis 2020;14:e0007975.
4. Bharadwaj S, Ginoya S, Tandon P, et al. Malnutrition: laboratory markers vs nutritional assessment. Gastroenterol Rep (Oxf) 2016;4:272-280.
5. Iqbal NT, Sadiq K, Syed S, et al. Promising Biomarkers of Environmental Enteric Dysfunction: A Prospective Cohort study in Pakistani Children. Sci Rep 2018;8:2966.
6. Kosek M, Haque R, Lima A, et al. Fecal markers of intestinal inflammation and permeability associated with the subsequent acquisition of linear growth deficits in infants. Am J Trop Med Hyg 2013;88:390-396.
7. Liu J, Kabir F, Manneh J, et al. Development and assessment of molecular diagnostic tests for 15 enteropathogens causing childhood diarrhoea: a multicentre study. Lancet Infect Dis 2014;14:716-724.
8. Liu J, Platts-Mills JA, Juma J, et al. Use of quantitative molecular diagnostic methods to identify causes of diarrhoea in children: a reanalysis of the GEMS case-control study. Lancet 2016;388:1291-301.
9. Platts-Mills JA, Liu J, Rogawski ET, et al. Use of quantitative molecular diagnostic methods to assess the aetiology, burden, and clinical characteristics of diarrhoea in children in low-resource settings: a reanalysis of the MAL-ED cohort study. Lancet Glob Health 2018;6:e1309-e1318.
10. Iqbal NT, Syed S, Kabir F, et al. Pathobiome driven gut inflammation in Pakistani children with Environmental Enteric Dysfunction. PLoS One 2019;14:e0221095.
11. Syed S, Yeruva S, Herrmann J, et al. Environmental Enteropathy in Undernourished Pakistani Children: Clinical and Histomorphometric Analyses. Am J Trop Med Hyg 2018;98:1577-1584.
12. Haberman Y, Karns R, Dexheimer PJ, et al. Ulcerative colitis mucosal transcriptomes reveal mitochondriopathy and personalized mechanisms underlying disease severity and treatment response. Nat Commun 2019;10:38.
13. Haberman Y, BenShoshan M, Di Segni A, et al. Long ncRNA Landscape in the Ileum of Treatment-Naive Early-Onset Crohn Disease. Inflamm Bowel Dis 2018;24:346-360.
14. Bray NL, Pimentel H, Melsted P, et al. Near-optimal probabilistic RNA-seq quantification. Nat Biotechnol 2016;34:525-7.
15. Chen J, Bardes EE, Aronow BJ, et al. ToppGene Suite for gene list enrichment analysis and candidate gene prioritization. Nucleic Acids Res 2009;37:W305-11.
16. Kaimal V, Bardes EE, Tabar SC, et al. ToppCluster: a multiple gene list feature analyzer for comparative enrichment clustering and network-based dissection of biological systems. Nucleic Acids Res 2010;38:W96-102.
17. Shannon P, Markiel A, Ozier O, et al. Cytoscape: a software environment for integrated models of biomolecular interaction networks. Genome Res 2003;13:2498-504.
18. Hart SN, Therneau TM, Zhang Y, et al. Calculating sample size estimates for RNA sequencing data. J Comput Biol 2013;20:970-8.

19. Haberman Y, Tickle TL, Dexheimer PJ, et al. Pediatric Crohn disease patients exhibit specific ileal transcriptome and microbiome signature. J Clin Invest 2014;124:3617-33.
20. Bragde H, Jansson U, Jarlsfelt I, et al. Gene expression profiling of duodenal biopsies discriminates celiac disease mucosa from normal mucosa. Pediatr Res 2011;69:530-7.
21. Zhang B, Horvath S. A general framework for weighted gene co-expression network analysis. Stat Appl Genet Mol Biol 2005;4:Article17.
22. Aryee MJ, Jaffe AE, Corrada-Bravo H, et al. Minfi: a flexible and comprehensive Bioconductor package for the analysis of Infinium DNA methylation microarrays. Bioinformatics 2014;30:1363-9.
23. Fortin JP, Labbe A, Lemire M, et al. Functional normalization of 450k methylation array data improves replication in large cancer studies. Genome Biol 2014;15:503.
24. Peters TJ, Buckley MJ, Statham AL, et al. De novo identification of differentially methylated regions in the human genome. Epigenetics Chromatin 2015;8:6.
